# Synthesis and Characterization of Biomimetic Thermoplastic Polyurethanes and Nanocomposites with l‑Lysine Diisocyanate

**DOI:** 10.1021/acs.biomac.5c01488

**Published:** 2025-12-01

**Authors:** Charlie Bateman, Chenghao Yao, Jingyang Lin, Shuai Zhang, Biqiong Chen

**Affiliations:** † Department of Chemistry, 4591 University of Liverpool, Crown Street, Liverpool L69 7ZD, U.K.; ‡ School of Mechanical and Aerospace Engineering, 1596 Queen’s University Belfast, Stranmillis Road, Belfast BT9 5AH, U.K.; § School of Pharmacy, Queen’s University Belfast, Lisburn Road, Belfast BT9 7BL, U.K.

## Abstract

Biomimetic materials are of significant interest in applications such as soft tissue repair, with their ability to replicate morphology and properties of native tissue. This study reports a novel thermoplastic polyurethane (TPU) synthesized with an amino acid-based diisocyanate hard segment. The effects of hard segment percentage on the mechanical, thermal, and hydrophilic properties were assessed. The optimal TPU shows a Young's modulus of 0.19 MPa, a tensile strength of 0.61 MPa, and an elongation at break of 2375%. Incorporating a novel functionalized clay in this TPU gives excellent antibacterial properties, demonstrating efficacy against both Gram-positive and Gram-negative bacterial strains. The addition of this clay also significantly enhances the mechanical properties of the TPU, with Young’s modulus increasing by up to 26 times with 3 wt % clay. The TPU was spun into fibers, creating a fibrous scaffold mimicking the architecture of some soft tissues. The TPU fibers exhibit a considerably higher tensile strength compared to bulk TPU while maintaining a high elongation at break. These TPUs and TPU–clay nanocomposites may find potential applications in soft tissue scaffolds or patches with antibacterial or anti-inflammatory behavior, for example, for the repair of gastrointestinal tissue that may be exposed to harmful bacteria.

## Graphical abstract

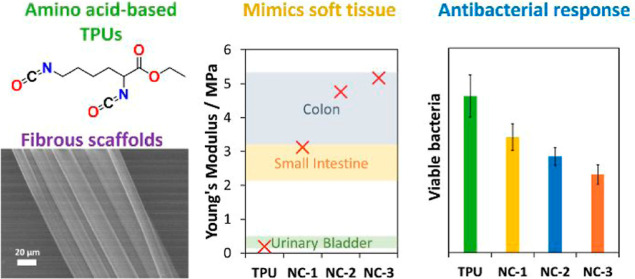

## 1 Introduction

Gastrointestinal cancers account for 1 in 4 cancer cases and 1 in 3 cancer deaths globally.^1^ The treatment of bowel cancers usually involves surgery to remove the cancer that often also removes a portion of healthy tissue and is sometimes not possible to reconnect the remaining tissue.^2^ When this is the case, a procedure called an ostomy is required that can develop into life-long conditions.^2^ Tissue engineering is a potential strategy for the reconstruction of the bowel, transplantation of autologous tissue, and treatment of gastrointestinal cancers.^3^ The advantages of this strategy are the scalability of production, tunability of properties, and consistency. Biomimetic scaffolds can imitate native tissue and influence cell growth depending on their structure, which reduces risk of rejection and can lead to more successful outcomes by allowing tissue to regrow throughout the structure.^4^ Young’s modulus for an adult human bowel tissue is around 2.7–5.2 MPa and for the colon is around 1.0–1.9 MPa.^5^ Disadvantages of tissue engineering include the risk of chronic inflammation and scaffold rejection.^6–9^ The scaffold for gastrointestinal tissue engineering would be exposed to harmful bacteria in the gastrointestinal system, and antibacterial functionality in the material would reduce the risk of these bacteria spreading through the structure, potentially causing damage to the surrounding tissue.

Thermoplastic polyurethanes (TPUs) are a type of materials that are commonly used in biomedical applications.^10–13^ The simplicity of modifying the chemical structure and properties through the selection of building blocks makes TPUs suitable candidates for a range of applications. The control of a TPU’s mechanical properties, biodegradability, and biocompatibility means it is possible to synthesize biomaterials suitable for the production of soft tissue scaffolds. Antibacterial functionality of the TPU may be controlled through the inclusion of specific functional groups^14^ or in the formation of a TPU nanocomposite (TPU-NC) with the addition of nontoxic nanofillers to enhance the desired properties of the polymer.

Nanoclays are widely used as reinforcement materials in nanocomposites because they improve mechanical and thermal properties without significantly increasing the material’s density and cost.^15^ Montmorillonite (MMT) is a commonly used aluminosilicate clay.^16,17^ Their high aspect ratio and large specific surface area give them excellent reinforcing capabilities, making them highly effective nanofillers. For example, exfoliated MMT sheets are about 1 nm thick, with lateral dimensions from around 50 nm to more than 1 μm and specific surface area of ca. 725 m^2^ g^–1^.^18–20^ In addition, MMT is nontoxic and has been widely used in biomedical applications.^21^

The overall aim of this research is to synthesize a novel, biobased TPU and antibacterial TPU-NCs for the potential use in the production of biomimetic and biocompatible patches and scaffolds with similar properties to those in the native bowel tissue. This will be achieved by using a poly­(ε-caprolactone) diol with a disulfide bond (PCL-DS), cyclohexane dimethanol (CHDM), and an amino acid-based diisocyanate, lysine diisocyanate (LDI), to prepare a TPU suitable for gastrointestinal tissue repair. Amino acid-based diisocyanates offer the resulting TPUs biodegradability and nontoxic degradation products while also contributing to reduced CO_2_ emissions and lower toxicity of diisocyanates.^22^ The impact of the hard segment (HS) ratio on properties will be analyzed to produce an optimal polymer for the preparation of fibrous TPU scaffolds to mimic the fibrous structure of gastrointestinal tissue and of TPU-NCs containing surface-functionalized MMT to enhance mechanical properties and incorporate antibacterial functionality. The chemical structure, thermal, mechanical, wettability, and antibacterial properties of the new TPUs and TPU-NCs will be investigated.

## 2 Experimental Section

### 2.1 Materials

ε-Caprolactone (ε-CL; 97%), hydroxyethyl disulfide (HEDS; technical grade), cyclohexane dimethanol (CHDM; mixture of cis and trans, 99%), phosphate buffered saline (PBS, tablets), tetrahydrofuran (THF, ≥99.9%, HPLC grade, inhibitor-free), diethyl ether (DEE, ≥99.0%, anhydrous, ACS reagent, BHT inhibitor), tin­(II) 2-ethylhexanoate (Tin­(II); 92.5–100%), and dibutyltin dilaurate (DBTDL; 95%) were purchased from Sigma-Aldrich. l-Lysine diisocyanate (LDI, 97%) was purchased from Alfa Aesar. Natural sodium MMT with the trade name of Cloisite Na^+^ and cation exchange capacity (CEC) of 92.6 meq. 100 g^–1^ was obtained from Southern Clay Products Inc., Texas, USA. 2-Undecylimidazoline (96%) was purchased from Doug Discovery. Eagle’s Minimum Essential Medium (MEM), fetal bovine serum, penicillin, MTT cell proliferation assay kit, and Triton X-100 were purchased from Thermo Fisher Scientific. *Escherichia coli* ATCC 25922 (*E. coli*), *Staphylococcus aureus* ATCC 29213 (*S. aureus*), and L929 mouse fibroblasts were purchased from the American Type Culture Collection.

### 2.2 Synthesis of TPUs

ε-CL and HEDS were melted and mixed at a 12:1 molar ratio in a 250 mL three-necked flask equipped with a condenser and nitrogen flow at 120 °C for 1 h. Subsequently, 0.1 wt % tin (II) was added and reacted for 24 h. Resultant PCL-DS was removed from the flask and vacuum-dried at 80 °C for 2 h in a vacuum oven held at 10 Pa and then left to cool for 24 h.

TPUs were synthesized in one step using a 100 mL round-bottom flask purged with nitrogen. To ensure all diol components were reacted with isocyanates, an NCO:OH ratio of 1.1:1 was used by dissolving PCL-DS, CHDM, and DBTDL (0.05 wt %) in THF and stirring at 60 °C for 1 h. Then, LDI was added and left to stir for 2 h. TPU solution was precipitated in DEE to remove remaining unreacted monomers and oligomers, poured, and evaporated overnight before drying in a vacuum oven at 30 °C for a further 24 h. TPUs had hard segment percentages of 40, 45, and 50 mol %, denoted as TPU-40, TPU-45, and TPU-50, respectively (see Table S1 for composition, Supporting Information). These hard segment contents are within the typical range for TPUs (10–50 mol %^23^) and the higher contents were selected to achieve stiffer TPUs^24^ for the targeted soft tissue repair.

### 2.3 Surface Functionalization of MMT

Cloisite Na^+^ was dispersed in distilled water for 3 days at 3 wt % concentration on a rolling table to ensure good dispersion.^25^ After this, the suspension was sonicated in a sonication bath for 30 min before being left to stand for 24 h, allowing impurities to settle to the bottom of the container and the supernatant was collected. For the functionalization of MMT, 2-undecylimidazoline was selected as the surfactant as it contains a long alkyl chain; antibacterial properties have been reported for molecules with long alkyl chains.^26,27^ A 5:1 volume ratio water:methanol solution was used to dissolve 2-undecylimidazoline. The clay supernatant was then mixed at 60 °C with a 100 mL solution containing 2-undecylimidazoline (92.6 mmol × 100 g^–1^) for 48 h in a sealed glass bottle. The suspension was stirred for about 24 h at room temperature (∼23 °C), left to settle for 24 h, and the supernatant was collected. Finally, the functionalized clay (denoted as MMT-U) was dried in a fume cupboard at room temperature for 72 h and then a vacuum oven at 60 °C for 24 h.

### 2.4 Preparation of TPU-NCs

TPU-NCs were produced by dissolving the selected TPU and dispersing a predetermined amount of MMT-U clay in a 5:1 THF:methanol solution for 24 h with stirring. TPU-NCs with concentrations of 1, 2, and 3 wt % MMT-U were prepared (denoted as NC-1, NC-2, and NC-3, respectively). These clay concentrations are in the typical range of clay loadings in polymer nanocomposites.^25^ After this first step, the solution was sonicated in a sonication bath for 60 min. The suspension was poured and evaporated overnight before drying in a vacuum oven at 30 °C for a further 24 h.

### 2.5 Preparation of TPU and TPU-NC Films for Characterization

TPU and TPU-NC films were produced using a hydraulic press with platens heated to 100 °C using a 0.5 mm thick steel mold. The material was left to melt for 2 min before being pressed for 5 min. The pressed films were cooled at room temperature for 24 h before being removed from the molds.

### 2.6 Preparation of TPU Fibers

Fiber spinning was carried out using a wet spinning method to produce TPU fibers. The TPU was dissolved in THF at a concentration of 12.5 wt %. Polymer solution was injected into a water bath at a 60° decline through a flat 17-gauge needle at 10 mL h^–1^. Fibers were drawn through the water bath to remove the THF and collected on an aluminum mandrel rotating at 100 rpm.

### 2.7 Characterization

Fourier transform infrared spectra (FT-IR) were obtained using a PerkinElmer Spectrum 2 in the range of 400–4000 cm^–1^ over 16 scans with a resolution of 4 cm^–1^. Nuclear magnetic resonance (NMR) was measured using a Bruker AVIII400 NMR Spectrometer with a CDCl_3_ mobile phase. Data was analyzed using TopSpin 3.6.3 software.

Gel permeation chromatography (GPC) was performed on an Agilent 1260 Infinity II GPC with Agilent GPC/SEC software and an RID detector using 2 × PLgel 5 μm MIXED-C columns (PS/DVB) and a 1 × PLgel 5 μm guard column at 35 °C and a flow rate of 1 mL min^–1^. Samples were prepared by dissolving the TPU in THF at a concentration of 3 mg mL^–1^ and filtered through a polytetrafluoroethylene syringe filter with a pore size of 0.45 μm. The GPC was calibrated using 12× EasiVial PS-H (2 mL) standards, with polystyrene molecular weights of 162, 580, 1210, 4880, 10,330, 22,790, 75,050, 194,500, 479,200, 885,000, 3,152,000, and 6,570,000 g·mol^–1^.

X-ray diffraction (XRD) patterns were recorded using a Malvern Panalytical Xpert Pro Multi-Purpose X-ray Diffractometer (model number DY1610) with Cu K_α_ irradiation (wavelength = 0.154 nm), a voltage of 45 kV, and a current of 40 mA, in the range of 4–65° 2θ with a step size of 0.017° and scanning speed of 1.7° min^–1^.

Differential scanning calorimetry (DSC) was carried out using a TA Discovery DSC25 with Trios v5 Software, and an aluminum pan with approximately 10 mg mass sample. All samples received three heat–cool cycles between −80 and 200 °C under a nitrogen flow (at a rate of 50 mL min^–1^), held for 2 min between cycles with a heating or cooling rate of 10 °C min^–1^. Thermogravimetric analysis (TGA) was run at 10 °C min^–1^ up to 600 °C under nitrogen flow (at a rate of 50 mL min^–1^) using the NETZSCH TG 209F1 Libra.

The surface water contact angle of TPU films (*n* = 3) was measured by using a Biolin Scientific Attension Theta Tensiometer. A droplet of water (20 μL) was placed onto the TPU film. Images were captured, and angles were measured using ImageJ software.

Uniaxial tensile tests of TPUs and TPU-NCs were performed on a Lloyds LRX with a 50 N load cell at a 100 mm min^–1^ strain rate until failure with a 0.01 N preload at ambient temperature. Samples and testing conditions were prepared according to ISO 37. Type 3 size dumbbell samples (*n* = 4, thickness: 0.45–0.6 mm) were cut from films using a die with dimensions specified in ISO 37. Uniaxial tensile tests of TPU fibers were performed on a Zwick/Roell z100 with a 20 N load cell at 10 mm min^–1^. Uniaxial cyclic tensile tests of TPUs and TPU-NCs were performed on a Lloyds LRX with a 50 N load cell at a 50 mm min^–1^ for a preconditioning cycle and 5 cycles at strains of 0–50% (*n* = 3, thickness: 1.8–2.1 mm). There was no resting time between cycles.

### 2.8 Cytotoxicity and Antibacterial Tests

Cytotoxicity assay was performed according to ISO 10993-5:2009. In brief, samples were first sterilized in 70% ethanol for 24 h at 4 °C, rinsed with sterile water, and air-dried in a biosafety cabinet. Conditioned medium was prepared by incubating each sample for 24 h in Eagle’s MEM supplemented with 10% fetal bovine serum and 1% penicillin. The conditioned medium was filtered using a sterile 0.2 μm filter and applied to L929 fibroblasts seeded at ∼1 × 10^5^ cells well^–1^. Cells were exposed to the conditioned media for 24 h at 37 °C (5% CO_2_), after which cell viability was measured by an MTT assay. Wells containing Triton X-100 and complete MEM served as the positive and negative controls, respectively.

Antibacterial tests were performed in a 96-well plate. Bacterial cultures of *E. coli* and *S. aureus* were cultured in Mueller Hinton Broth overnight and diluted in phosphate buffered saline (PBS) to achieve a concentration of 5 × 10^5^ CFU mL^–1^. Ultraviolet (UV) sterilized samples were placed into designated wells in the 96-well plate and 20 μL of bacterial suspension was added to the surface of each sample. Samples were gently removed from wells using sterile forceps and washed with sterile PBS to remove loosely attached bacteria. Washed samples were transferred into a new plate, and 200 μL of sterile PBS was added to each sample well. The plate was placed in a sonication bath for 20 min to detach bacteria from the sample surface. After sonication, 20 μL of the PBS solution was taken from each well and transferred to 180 μL of sterile PBS in a new well. This was repeated twice to achieve a 100-fold dilution. Ten μL was taken from the final dilution and spread onto Mueller–Hinton Agar (MHA) plates before being incubated overnight at 37 °C. Colonies were counted on the MHA plates to determine the number of viable bacteria.

## 3 Results and Discussion

### 3.1 Characterization of TPUs

#### 3.1.1 Structure of TPUs

PCL-DS was first synthesized by ring-opening polymerization of ε-CL with HEDS (Scheme 1). The resultant PCL-DS shows a number-average molecular weight ( ${\mathrm{M̅}}_{n}$ ) of 2500 g·mol^–1^ and polydispersity index (PDI) of 1.5, as determined by GPC (Figure S1, Supporting Information).

**Scheme 1:**
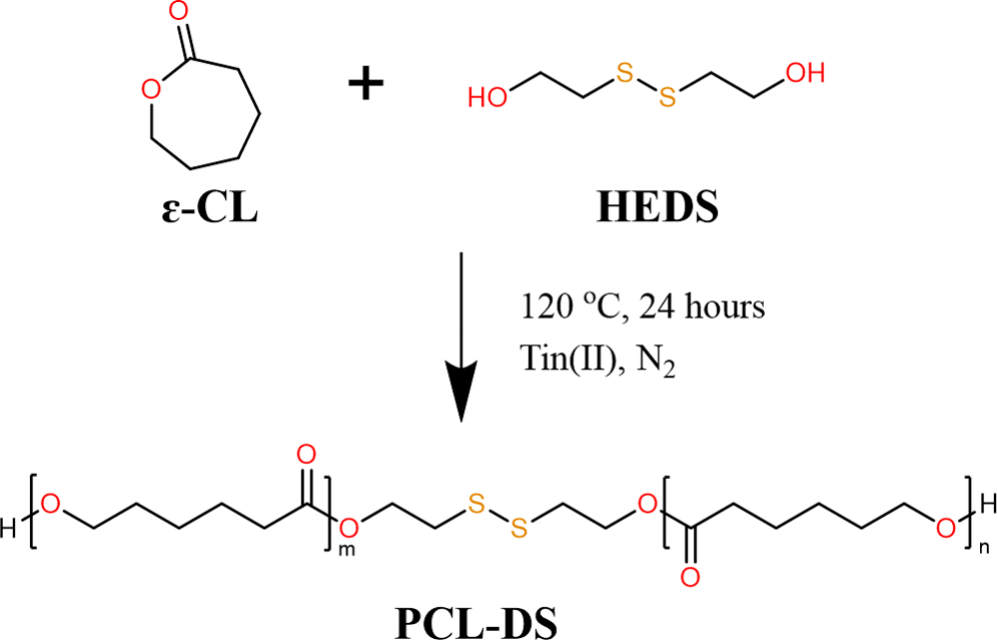
Synthesis of PCL-DS

To prepare TPUs, PCL-DS, as the soft segment, was mixed with the chain extender CHDM, followed by polymerization with the hard segment, LDI, through the reaction of hydroxyl groups in the two diols with the isocyanate groups in LDI (Scheme 2). By adjusting the molar ratios of these components, three TPUs with 40 mol %, 45 mol %, and 50 mol % hard segments were synthesized.

**Scheme 2:**
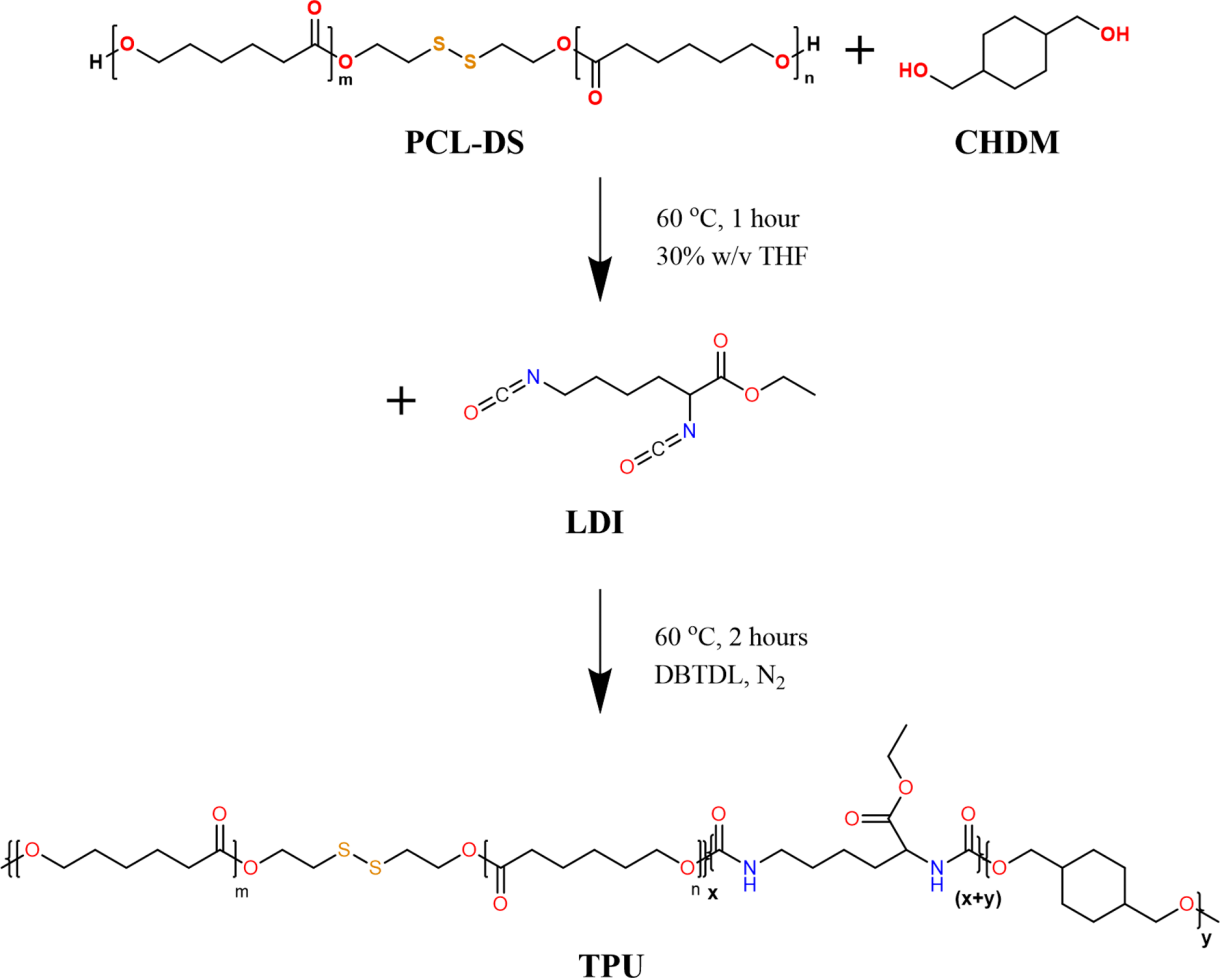
Synthesis of TPUs

FTIR was used to characterize the chemical structure of the resulting TPUs (Figure 1). N–H and C=O, C–O, or ester groups confirm the formation of urethane bonds. Primary, secondary, and tertiary C–O stretching alcohol groups were found at 1045 cm^–1^, 1095 cm^–1^, and 1158 cm^–1^, respectively.^28,29^ Ester groups occur at 1239 and 1722 cm^–1^. An aliphatic N–H stretching primary amine is present at 3350 cm^–1^. These characteristic peaks are associated with polyurethanes. There is also absence of a peak between 2250 and 2275 cm^–1^, which corresponds to isocyanate groups.^30,31^ This absence confirms that the toxic isocyanate groups in the LDI have fully reacted due to the use of excess OH groups. Other notable peaks observed in the spectra include aliphatic CH_2_ groups at 1454 cm^–1^, 2864 cm^–1^, and 2934 cm^–1^. The FTIR analysis confirms the successful synthesis of the TPUs with the expected urethane bonds, similar spectra for all TPUs, and the complete reaction of isocyanate groups, ensuring a nontoxic polymer structure.

**Figure 1:**
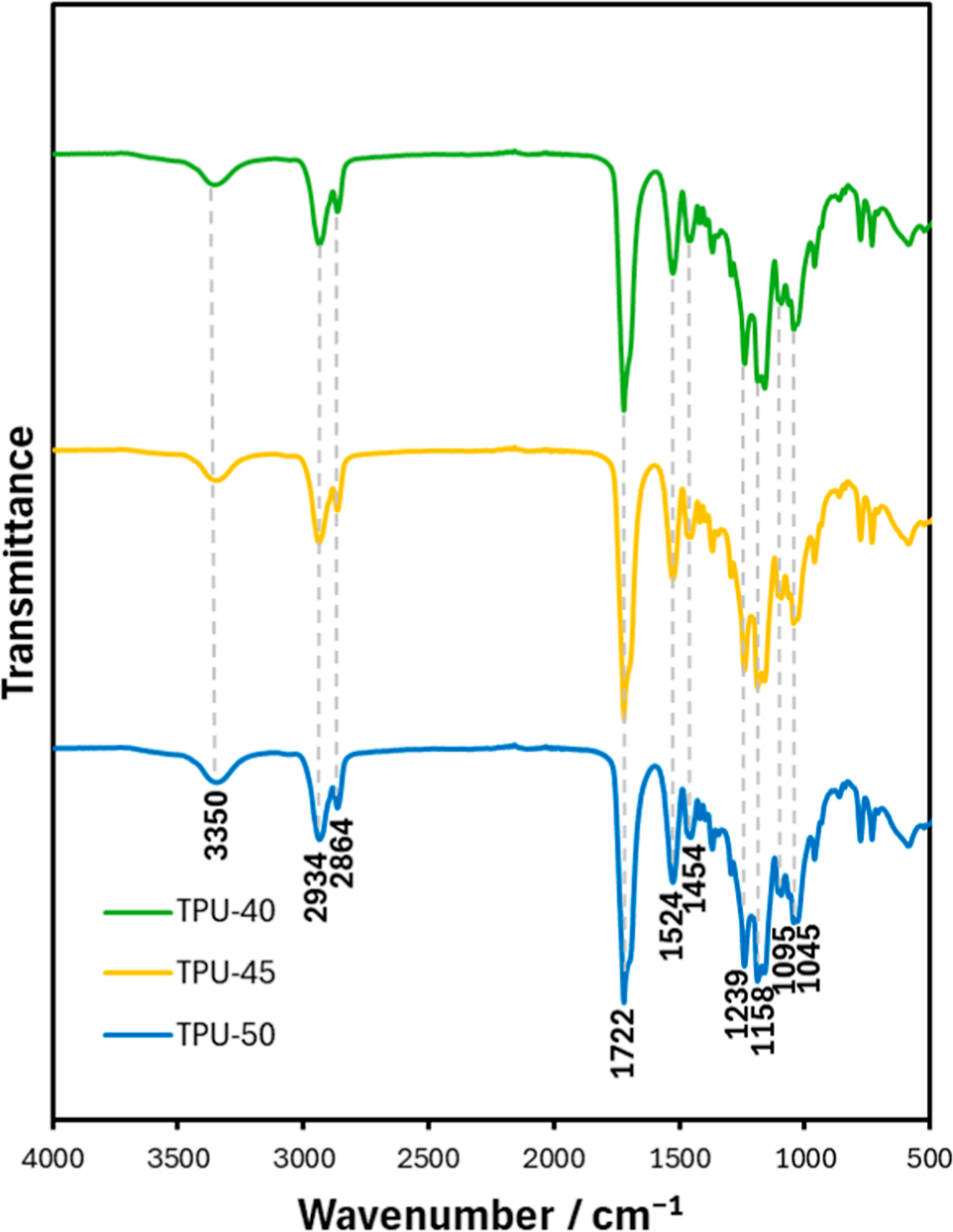
FTIR spectra of TPUs—spectra were shifted vertically for clarity.

^13^C and ^1^H NMR spectroscopy was used to further confirm the formation of TPUs. Chemical shifts associated with the TPUs are shown in Figure 2 and the similar peaks show the same components being used in all TPUs. ^13^C NMR spectra show the carbons from ester bond C=O at 173.5 ppm (peak 1), which is characteristic of carbonyl groups in the urethane linkages.^32^ The carbon of the C–O bond becomes apparent at 64.2 ppm (peak 2). At 14.2 and 34.1 ppm, primary alkyl carbons (CH_3_) are shown and a secondary alkyl carbon is detected at 28.4 ppm (peaks 3, 4, and 5).^33,34^ ^1^H NMR spectra show the amine proton of the urethane bond is visible at 3.2 ppm in ^1^H NMR^35^ (peak 10). The ester proton causes peaks at 4.1 ppm, CH_2_ protons are at 2.3 ppm, and CH_3_ protons are seen at 1.7 ppm^34^ (peaks 6, 7, and 9).

**Figure 2:**
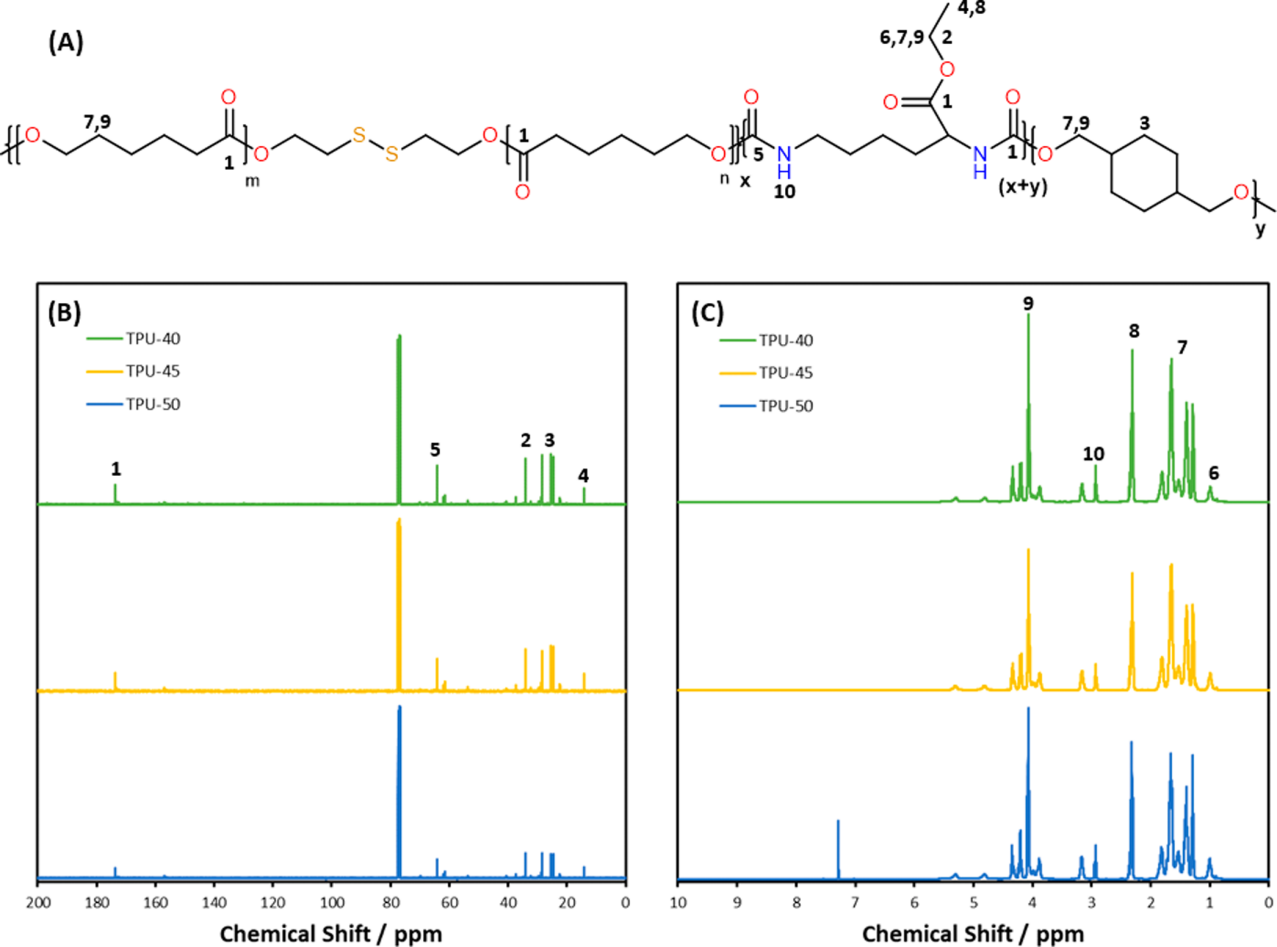
(A) NMR peak locations, (B) ^13^C NMR spectra, and (C) ^1^H NMR spectra for TPUs.

GPC results (Figure 3) show that TPU-40, TPU-45, and TPU-50 have ${\mathrm{M̅}}_{n}$ values of 188,500, 233,100, and 211,700 g·mol^–1^, respectively, and weight-average molecular weights ( ${\mathrm{M̅}}_{w})$ of 596,600, 754,900, and 734,500 g·mol^–1^, respectively. These values resulted in PDIs of 3.16, 3.23, and 3.46 for TPU-40, TPU-45, and TPU-50, respectively. This result implies a relatively high-molecular-weight distribution for the TPUs, caused by the side ethyl ester chains present in the LDI, and a wider molecular weight distribution with an increasing hard segment ratio. The small peaks below Log *M* of 4 account for trace amounts of unreacted monomers and oligomers, despite the precipitation purification step following polymerization.

**Figure 3:**
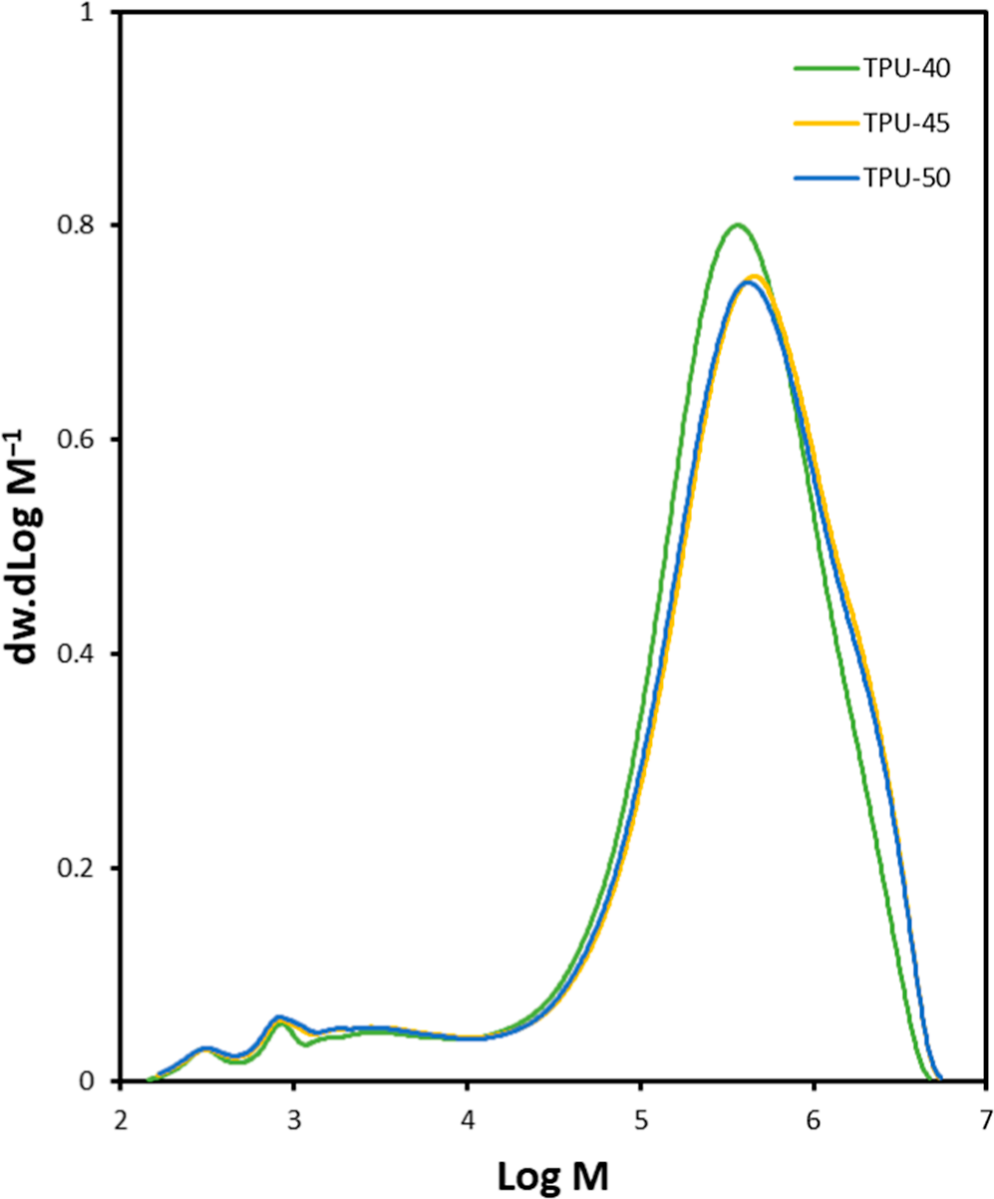
GPC curves of TPUs.

#### 3.1.2 Thermal Properties of TPUs

Thermal properties of TPUs were investigated using DSC and TGA. The midpoint glass transition temperatures (*T*_g_) were −33.6 °C, −29.1 °C, and −24.3 °C for TPU-40, TPU-45, and TPU-50, respectively (Figure 4). These low *T*_g_ values confirm that the TPUs have a flexible and rubbery state at room temperature. *T*_g_ increases with the hard segment ratio, in line with the literature.^36^ The lack of melting peaks within the DSC curves shows that the TPUs are amorphous. Due to their uncross-linked chain structure, the TPUs are thermoplastic and can gradually soften and flow for melt processing as the temperature rises to significantly higher than *T*_g_. The asymmetrical structure of the LDI with a methyl side chain can contribute to a lack of crystalline regions being formed in the TPUs.^37^

**Figure 4:**
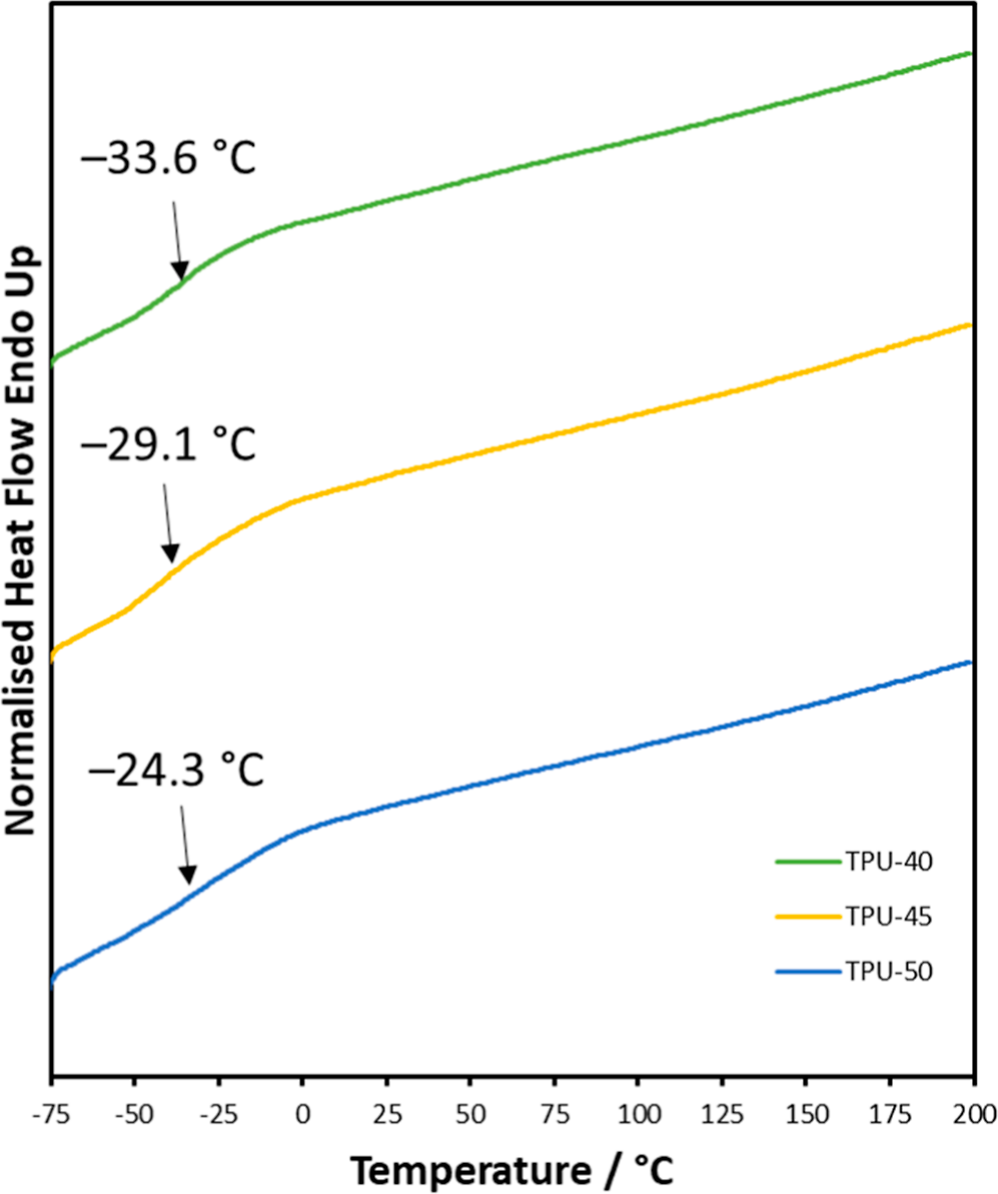
DSC third heating curve for TPUs—curves were shifted for clarity.

TGA curves of TPUs show at 100 °C an initial mass loss between 0.8 and 1.0% caused by the removal of moisture from the TPUs (Figure 5A). Degradation of TPU-40 starts at 205 and 230 °C for TPU-45 and TPU-50, respectively; all TPUs present a shoulder at 290 °C caused by the breakdown of the urethane bonds.^38^ The next step is the thermal degradation (*T*_d_) of the hard segment, with TPU-40, TPU-45, and TPU-50 reaching peak degradation temperature (*T*_d_^peak^) at 325 °C, 345 °C and 360 °C, respectively, in Figure 5B. The increase in *T*_d_^peak^ correlates to the increase in the hard segment ratio improving the thermal stability as a result of the cyclohexane ring.^36^ The final step is the degradation of the remaining PCL-DS chain between 390 and 460 °C.^39^ At 600 °C, a residue of 3.0–3.5% remains. This excellent thermal stability ensures melt processing of the TPUs into desired forms and shapes without causing degradation.

**Figure 5:**
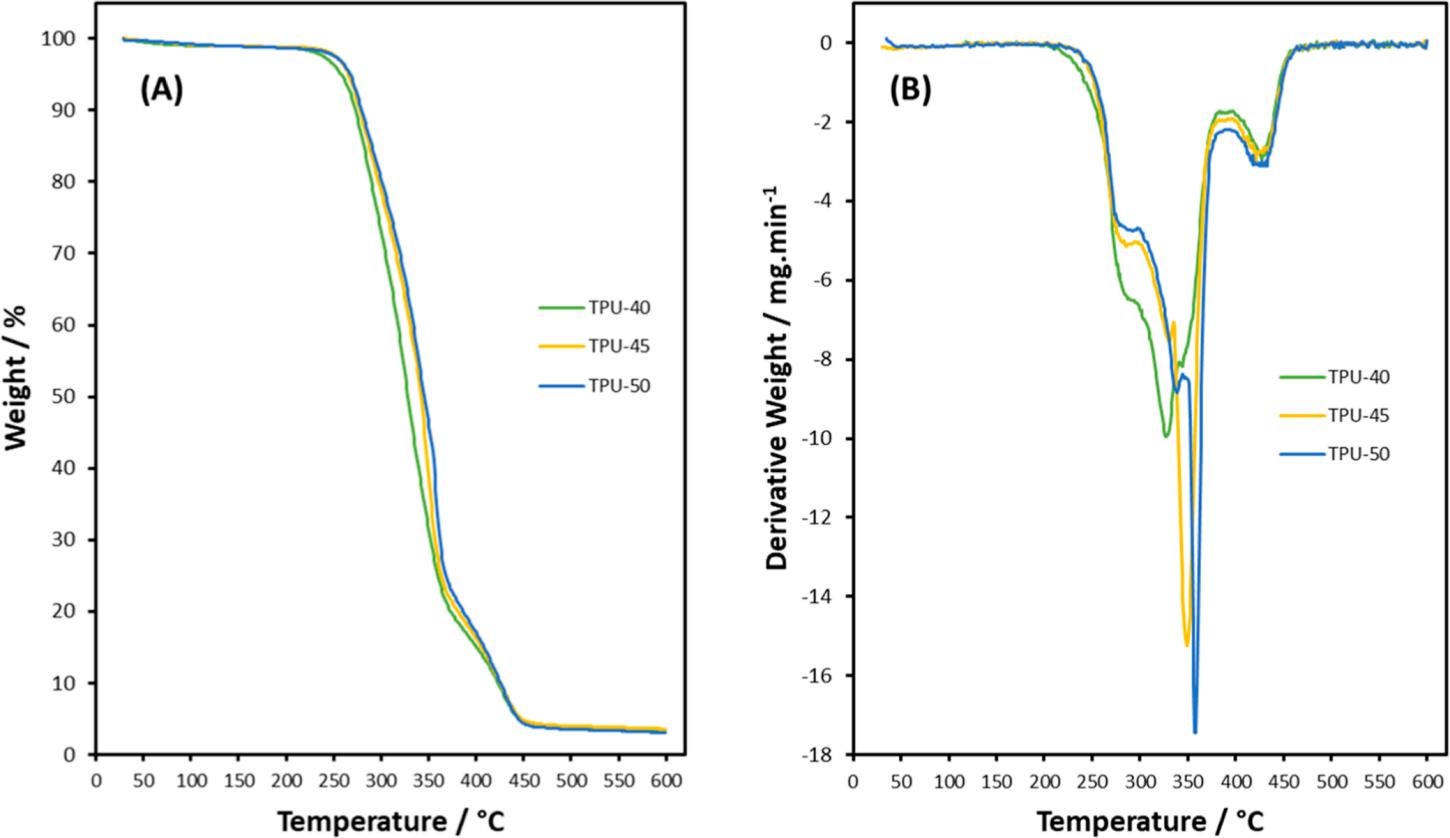
(A) TGA and (B) DTG curves for TPUs.

#### 3.1.3 Mechanical Properties of TPUs

Representative tensile curves of TPUs are shown in Figure 6A. Each TPU exhibits a steep linear elastic region before yielding, after which tensile stress causes the molecular chains to slide past each other, causing irreversible changes in plastic deformation. After a point, the force applied to deform the TPU increased and led to strain hardening until failure, attributable to good chain alignment^40^.

**Figure 6:**
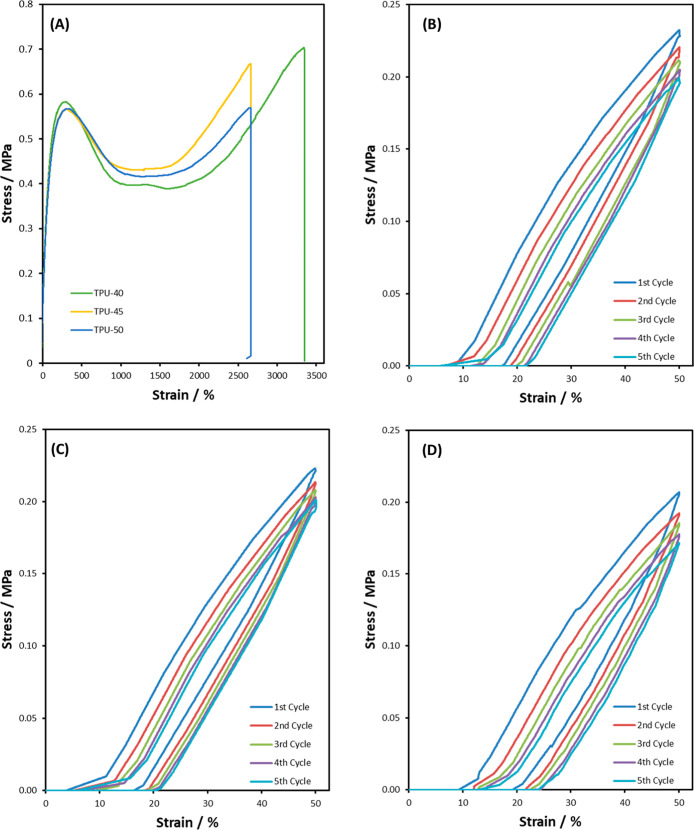
(A) Representative tensile stress–strain curves for TPUs and (B–D) cyclic tensile curves of TPU-40, TPU-45, and TPU-50 for 5 cycles after a preconditioning cycle.

The results from the tensile testing are summarized in Table 1. Young’s modulus (*E*) remained similar values, ranging from 0.18 to 0.20 MPa, with no statistical significance (two-tailed *t*-test, confidence interval *p* <0.05) despite variations in hard segment content. Similarly, the yield stress (σ_*y*_) stayed in the range of 0.56 to 0.58 MPa, also without statistical significance. The yield strain (ε_*y*_) increased with the hard segment percentage, rising from 286.5 ± 19.2% for TPU-40 to 319.6 ± 12.2% for TPU-50. The tensile strength (σ_max_) remained similar across the different compositions with values between 0.60 and 0.65 MPa and no statistical significance was observed.

**Table 1:** Tensile Properties of TPUs

sample	*E*/MPa	σ_y_/MPa	ε_y_/%	σ_max_/MPa	ε_max_/%
TPU-40	0.20 ± 0.02	0.58 ± 0.03	286.5 ± 19.2	0.65 ± 0.10	3200 ± 195
TPU-45	0.19 ± 0.01	0.56 ± 0.04	302.3 ± 29.1	0.61 ± 0.08	2375 ± 215
TPU-50	0.18 ± 0.01	0.57 ± 0.02	319.6 ± 12.2	0.60 ± 0.05	2475 ± 168

TPU-40 had the lowest hard segment percentage but gave the highest strain at break (ε_max_) at 3200 ± 195%. TPU-50 having the highest hard segment percentage exhibited a lower strain at a break of 2475 ± 168%. TPU-45 with an intermediate hard segment percentage showed a strain at break similar to TPU-50 at 2375 ± 215%. These high elongation at break values are likely from the high chain entanglement within the polymer chains resulting from their high molecular weights.^41^

Cyclic tensile stress strain curves for prestretched TPU-40, TPU-45, and TPU-50 are in Figure 6B, C, and D showing 5 loading cycles up to 50% strain at a rate of 50 mm min^–1^ with no rest time between cycles. The zero-stress plateau present in the initial stages of the loading cycle is a result of the TPU having not fully recovered from the previous cycle partly due to the high-speed testing. Hysteresis ratio (*h*_r_) was calculated according to eq 1, with e_0_ and e_r_ representing the input and retraction strain energies from the loading and unloading curves, respectively.

1 $$h_{r}=\frac{e_{o}-e_{r}}{e_{0}}$$

*h*_r_ of TPU-40, TPU-45, and TPU-50 for cycle 1 was 0.33, 0.32, and 0.31, respectively, and 0.34, 0.32, and 0.30 after 5 cycles respectively, implying a consistent loss of energy during each loading cycle, after a preconditioning cycle. There is a small drop in stress over each cycle due to the lack of rest time; the polymer does not have sufficient time to recover fully to its original state.

#### 3.1.4 Hydrophilicity of TPUs

Water contact angles of TPUs are shown in Figure 7. All TPUs can be classified as hydrophilic with contact angles below 90°.^42^ TPU-40, TPU-45, and TPU-50 became increasingly hydrophilic with the increase in hard segment (LDI) at 87.7 ± 2.8°, 82.2 ± 0.5°, and 80.4 ± 1.3°, respectively. The positive charge in the repeating hydrophilic lysine component of the chain causes a reduction in the contact angle, meaning an increase in surface wettability. This surface property will be an important factor regarding cell adhesion to the TPU.^43^

**Figure 7:**
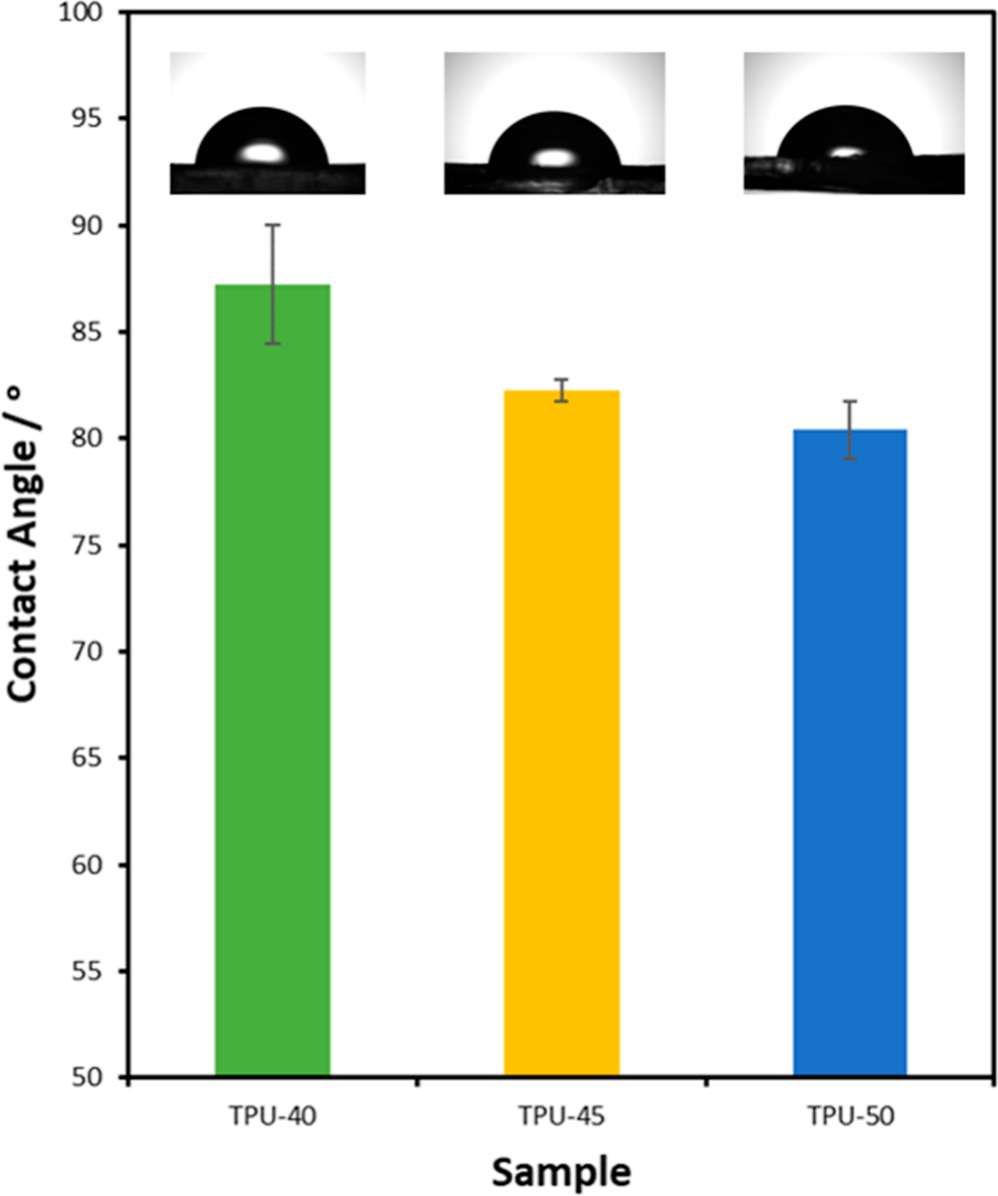
Water contact angle of TPUs.

As it provided an optimal balance of mechanical properties and hydrophilicity, TPU-45 was selected as the polymer to be used in the production of TPU nanocomposites (TPU-NCs) with MMT-U and TPU fibers in subsequent sections.

### 3.2 Characterization of Functionalized Clay

To improve the mechanical performance of TPU-45 and add antibacterial functionality, the addition of functionalized clays was explored. Modifying the surface of MMT clay is a strategic approach to introduce specific functional groups and surface functionality to the clay to improve existing properties such as mechanical strength and stiffness^44^ and/or incorporate new functionality, such as cell adhesion^25^ or antibacterial activity.^45^ 2-Undecylimidazoline was selected in the surface functionalization due to its long hydrophobic tail being capable of high levels of antibacterial functionality.^46^

Changes to the chemical structure of MMT clay can be observed through changes in the FTIR spectra shown in Figure 8. All bands in the range of 3100–3700 cm^–1^ are due to O–H stretching vibrations with structural hydroxyl groups and interlayer water molecules in clay and 1600–1700 cm^–1^ shows OH bending vibration bands within the clay structure. A Si–OH stretching band is detected at 971 cm^–1^. The large peak at 980 cm^–1^ indicates the presence of highly condensed siloxane (Si–O–Si).^47,48^ MMT-U shows a peak present at 3619 cm^–1^ representing the O–H groups present in the MMT clay. The long methylene chain present in MMT-U produces a prominent CH_2_ peak at 1469, 2848, and 2923 cm^–1^. The sharp peak at 1620 cm^–1^ in MMT-U represents the imidazole ring.^49^

**Figure 8:**
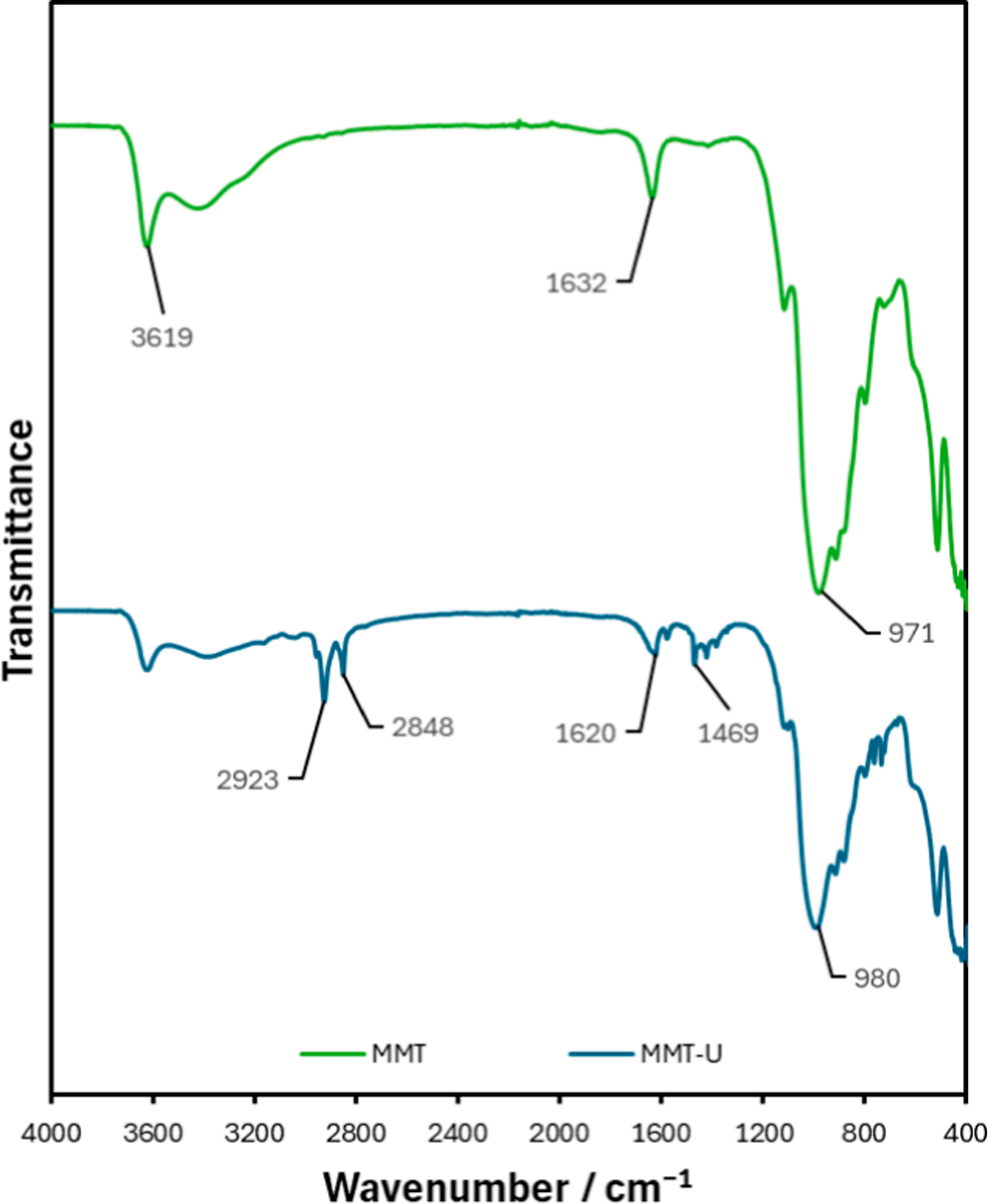
FTIR spectra of MMT and functionalized MMT clays—spectra were shifted vertically for clarity.

A well-established method for the characterization of clays and functionalized clays is the use of XRD (Figure 9). This allows for the expansion of clay galleries to be determined. The *d*-spacing, *d*_(001)_, of the unmodified MMT is 1.24 nm calculated from the reflection at 2θ = 7.15° using Bragg’s equation.^50^ After the cation exchange reaction, the (001) position of the clay shifts to a lower position at 6.37° for MMT-U resulting in a *d*_(001)_ of 1.39 nm. The increase in the distance between layers confirms that surface modification and intercalation of 2-undecylimidazoline have been successful. The increased *d*_(001)_ values are close to the values for the monolayer conformation of organic compounds in clay galleries.^51^

**Figure 9:**
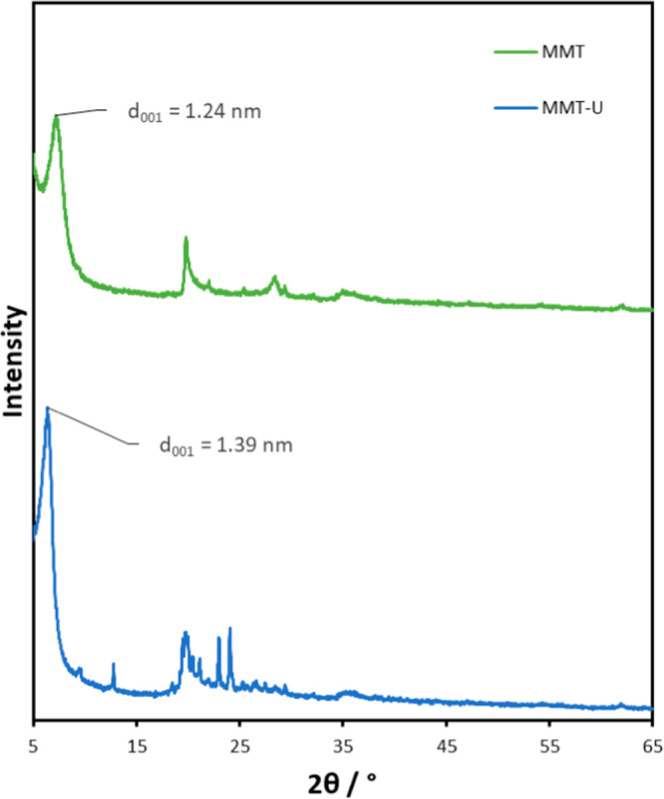
XRD spectra of modified MMT clay—spectra were shifted vertically for clarity.

### 3.3 Characterization of TPU Nanocomposites

Three nanocomposites with 1, 2, and 3 wt % MMT-U (NC-1, NC-2, and NC-3) were produced using TPU-45 (denoted as TPU from herein).

Figure 10 demonstrates the FTIR spectra of TPU-NCs with their control samples, MMT-U clay and TPU. The Si–O–Si peak in the nanocomposites was observed at 1026 cm^–1^, an increase from its original position of 994 cm^–1^ in MMT-U. This increase indicates a distortion in the Si–O–Si bond angle, which is a result of molecular confinement in the TPU by hydrogen bonding between the OH or C=O of TPU and the Si–O–Si or Si–OH of MMT-U.^25^ This peak becomes more apparent in the NC samples with an increased clay content. Another apparent change of the FTIR spectrum of the nanocomposites compared to TPU is the increased intensity of primary alcohol peaks at 1063 cm^–1^ and amine peaks at 1043 cm^–1^,^52–54^ as a result of the presence of hydroxyl groups and the imidazole ring in the MMT-U, respectively.

**Figure 10:**
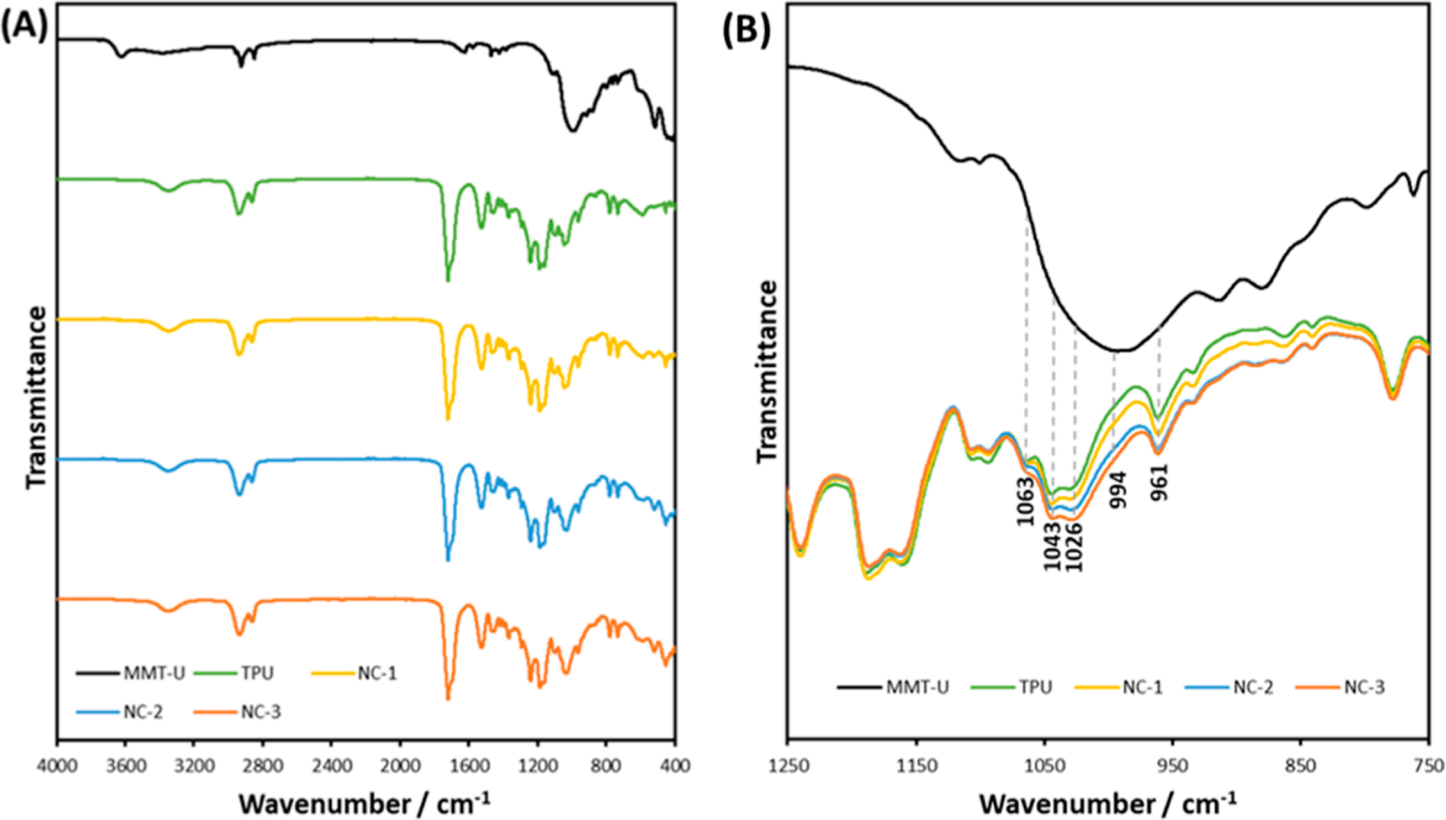
(A) FTIR spectra for MMT-U, TPU, and NCs and (B) focused view showing increased hydrogen bonding with increased clay loading—spectra were shifted vertically for clarity.

Representative tensile curves of TPU-NCs are shown in Figure 11 and the tensile properties are summarized in Table 2. As the TPU and NCs are stretched, the polymer chains will uncoil and align before reaching a yield point; the MMT-U in the NCs restricts this movement and requires more force, ultimately leading to higher stress values. The steep linear elastic region compared to that of the pristine TPU will result in a significantly higher Young’s modulus, allowing for the materials to have application in a wider range of soft tissue types that require higher stiffness. As shown in Figure 11A and Table 2, all TPU-NCs exhibit a considerable increase in Young’s modulus when compared to the pristine TPU by 1550%, 2400%, and 2620% for NC-1, NC-2, and NC-3, respectively, due to the high modulus, aspect ratio, and large specific surface area of the nano reinforcement.^44^ The same was true for the tensile strength at 640%, 800%, and 600% increases for NC-1, NC-2, and NC-3, respectively, suggesting the load from the polymer chains was effectively transferring to the rigid clay^55^ due to the strong hydrogen bonding between the functionalized clay and the TPU matrix. When the MMT-U clay is incorporated into the TPU, the yield strain decreases significantly. In comparison, the strain at break slightly increases at lower clay concentrations from 2374 ± 215% for pristine TPU to 2758 ± 121% for NC-2. At the highest concentration investigated (NC-3), the strain at break decreases to 1856 ± 410%, which may be attributable to the formation of some clay agglomerates in the nanocomposite, causing stress concentration points that restrict chain mobility.^56^

**Figure 11:**
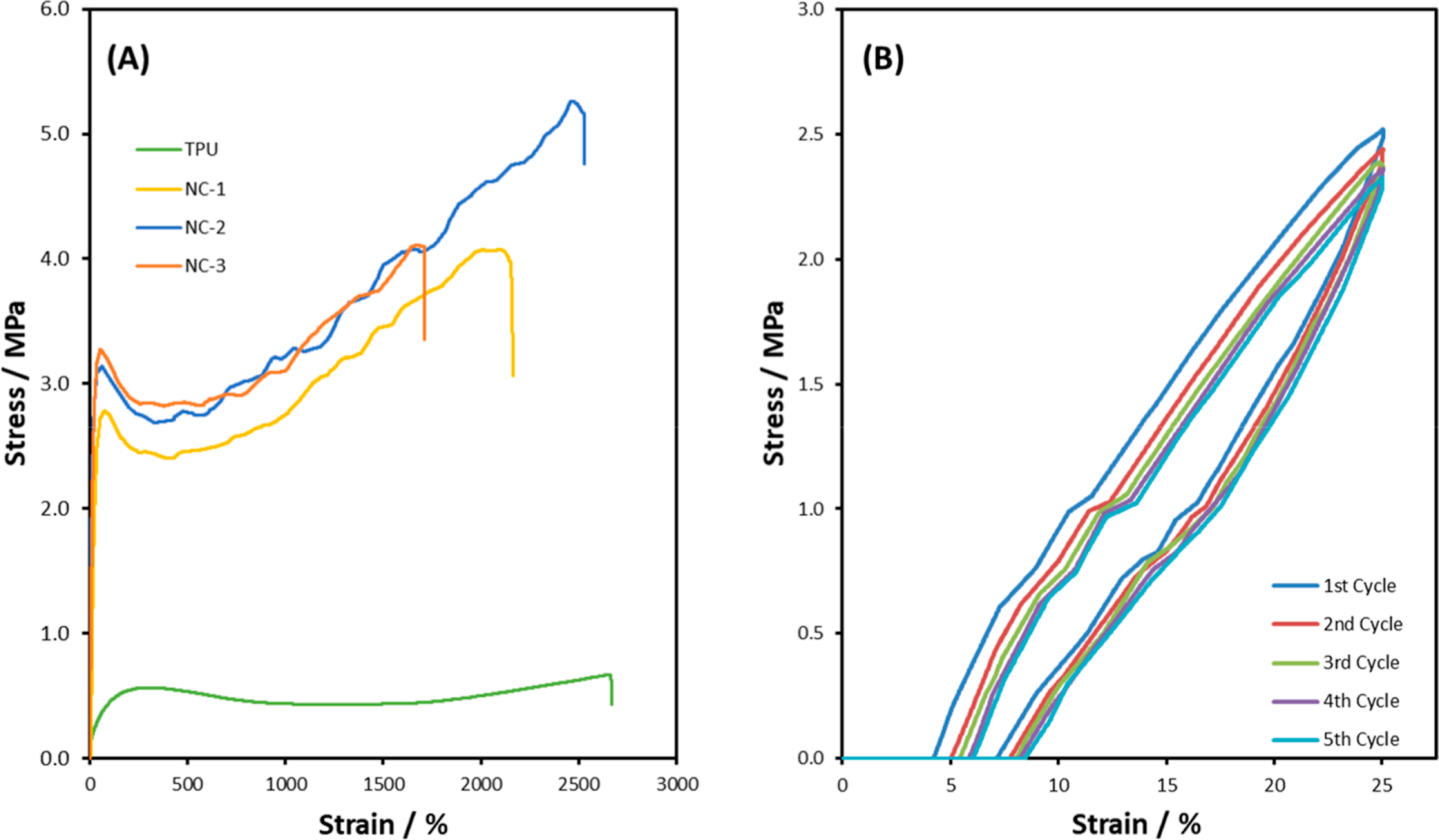
(A) Representative tensile stress–strain curves for TPU-NCs and (B) cyclic tensile curves of NC-2 for 5 cycles after a preconditioning cycle.

**Table 2:** Tensile Properties of TPU-NCs

sample	E/MPa	σ_y_/MPa	ε_y_/%	σ_max_/MPa	ε_max_/%
TPU	0.19 ± 0.01	0.56 ± 0.04	302.3 ± 29.1	0.61 ± 0.08	2374 ± 215
NC-1	3.12 ± 1.15	2.29 ± 0.70	82.3 ± 17.3	4.53 ± 0.37	2490 ± 387
NC-2	4.76 ± 0.70	2.99 ± 0.27	62.5 ± 6.1	5.50 ± 0.37	2758 ± 121
NC-3	5.17 ± 0.77	3.22 ± 0.36	71.6 ± 10.0	4.25 ± 0.38	1856 ± 410

Cyclic tensile stress–strain curves for NC-2 (the optimal TPU-NC) are in Figure 11B showing five loading cycles up to 25% strain at a rate of 50 mm min^–1^ with no rest time between cycles after a preconditioning cycle. Hysteresis ratio of NC-2 after 1 and 5 cycles was 0.50 and 0.48, respectively, showing a similar loss of energy during each loading cycle between the cycles but higher when compared to the pristine TPU. Hysteresis is often higher in nanocomposites due to the nanoscale interactions and microstructure causing additional energy dissipation during deformation.^57^

Water contact angles of TPU-NCs are shown in Figure 12, which are 78.4 ± 0.1°, 80.5 ± 0.9°, and 82.8 ± 0.4°, respectively. Compared to the pristine TPU (82.2 ± 0.5°), the contact angle reduces slightly for the NCs with lower clay contents, then level off at NC-3. MMT is naturally hydrophilic. When it is functionalized with 2-undecylimidazoline, which contains a hydrophilic amine group in the imidazole ring and a long hydrophobic alkyl tail, to become MMT-U, both its hydrophilicity and interactions with the TPU vary. At the higher clay contents, the hydrophobic effect from the alkyl tail becomes more dominant, leading to higher contact angles for the NCs.

**Figure 12:**
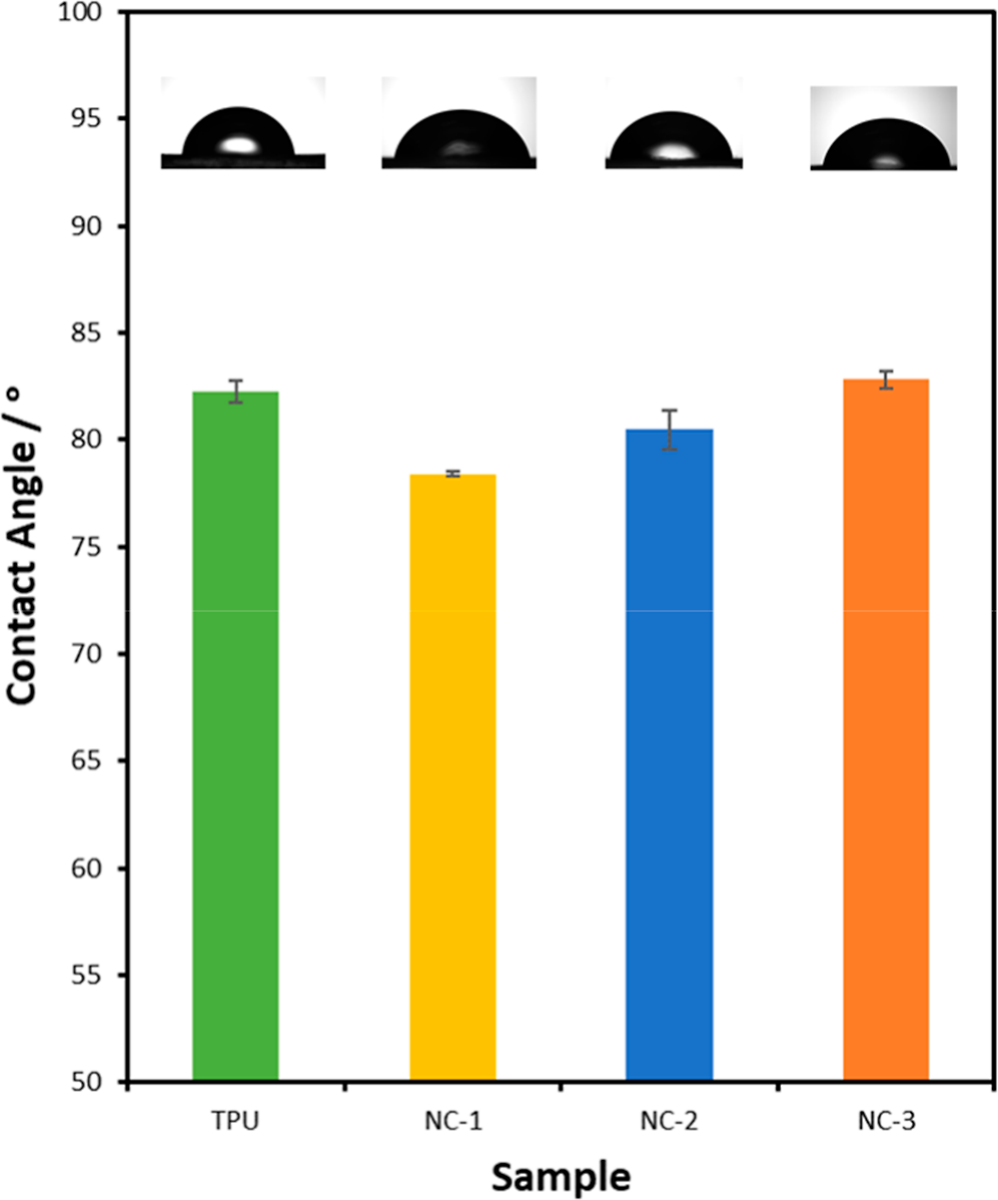
Water contact angle of TPU-NCs.

### 3.4 Potential Applications in Soft Tissue Engineering

#### 3.4.1 Mechanical Properties

The TPU and TPU-NCs synthesized show promise in a variety of soft tissue in the gastrointestinal and urinary systems. Each material shows Young’s modulus within the range of different organs (Figure 13). Pristine TPU has a Young modulus comparable to that of the urinary bladder, NC-1 is similar to the small intestine and NC-2 and NC-3 show similarity to the colon.^5^ Similar Young’s modulus values are crucial, as they reduce the likelihood of delamination during use. These biomimetic mechanical properties offer significant potential in tissue repair by replicating the native tissue environment and directing cell regeneration.^58^

**Figure 13:**
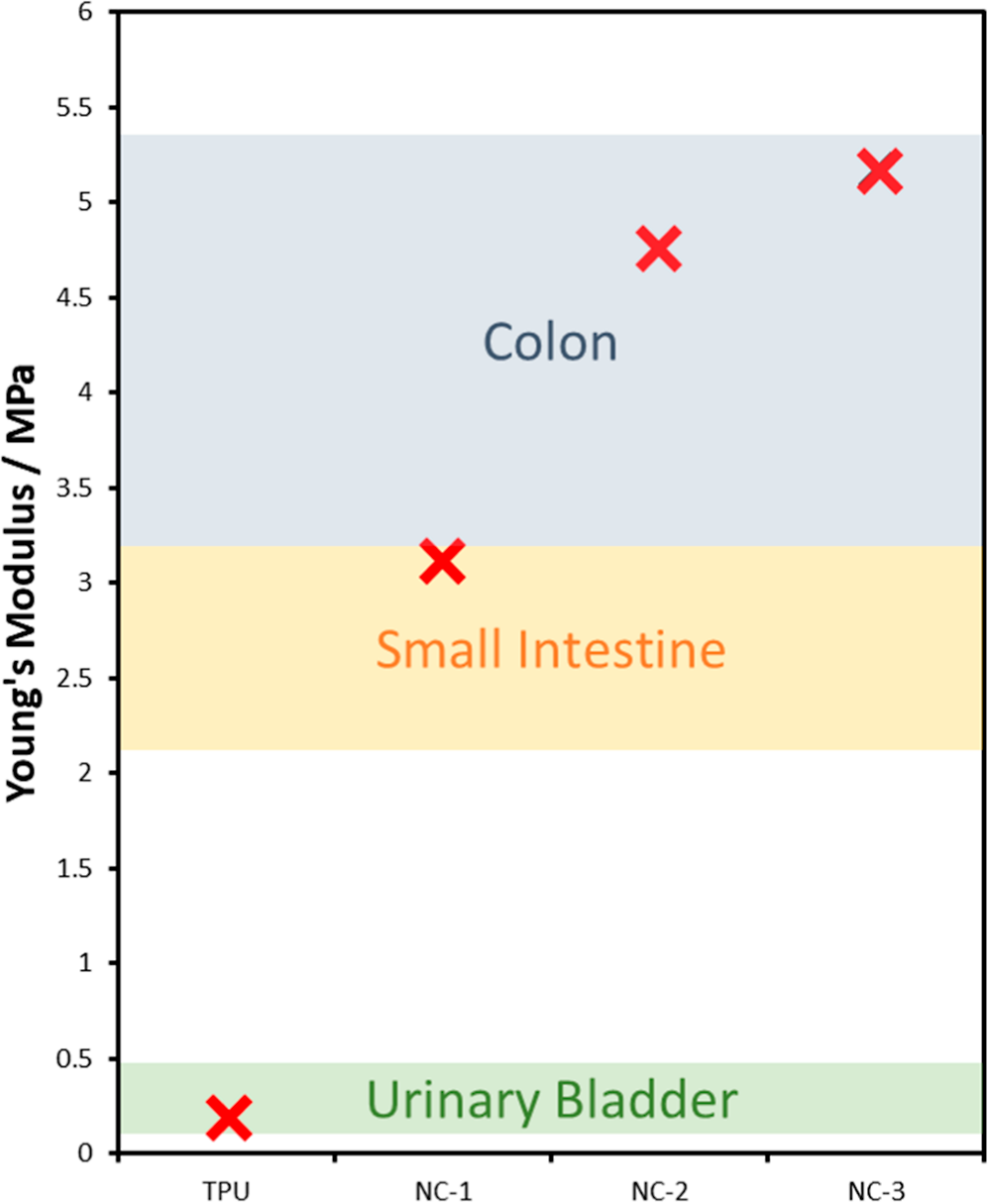
Comparison on Young’s modulus of TPU and TPU-NCs to soft tissue^5^.

#### 3.4.2 Cytocompatibility

Cytocompatibility is an important property for tissue repair. TPU, NC-1, NC-2, and NC-3 were analyzed to assess the cell viability and to determine if they had a cytotoxic effect on living cells. Figure 14 shows the cells remained 91.2 ± 2.0%, 89.2 ± 2.8%, 87.7 ± 3.4%, and 85.5 ± 3.6% viable after 24 h. While there are minor decreases in cell viability with the addition of MMT-U, the classification outlined in ISO 10993-5 states that cell viability of over 70% is considered nontoxic.^59^ The reduction of NC-1 and NC-2 relative to the TPU is not statistically significant; however, NC-3 has a minor reduction with statistical significance relative to the TPU (*p* <0.05). 2-Undecylimidazoline’s hydrophobic chain structures intended for adding antibacterial functionality to the NCs might have been the cause of such minor reduction.^26,27^

**Figure 14:**
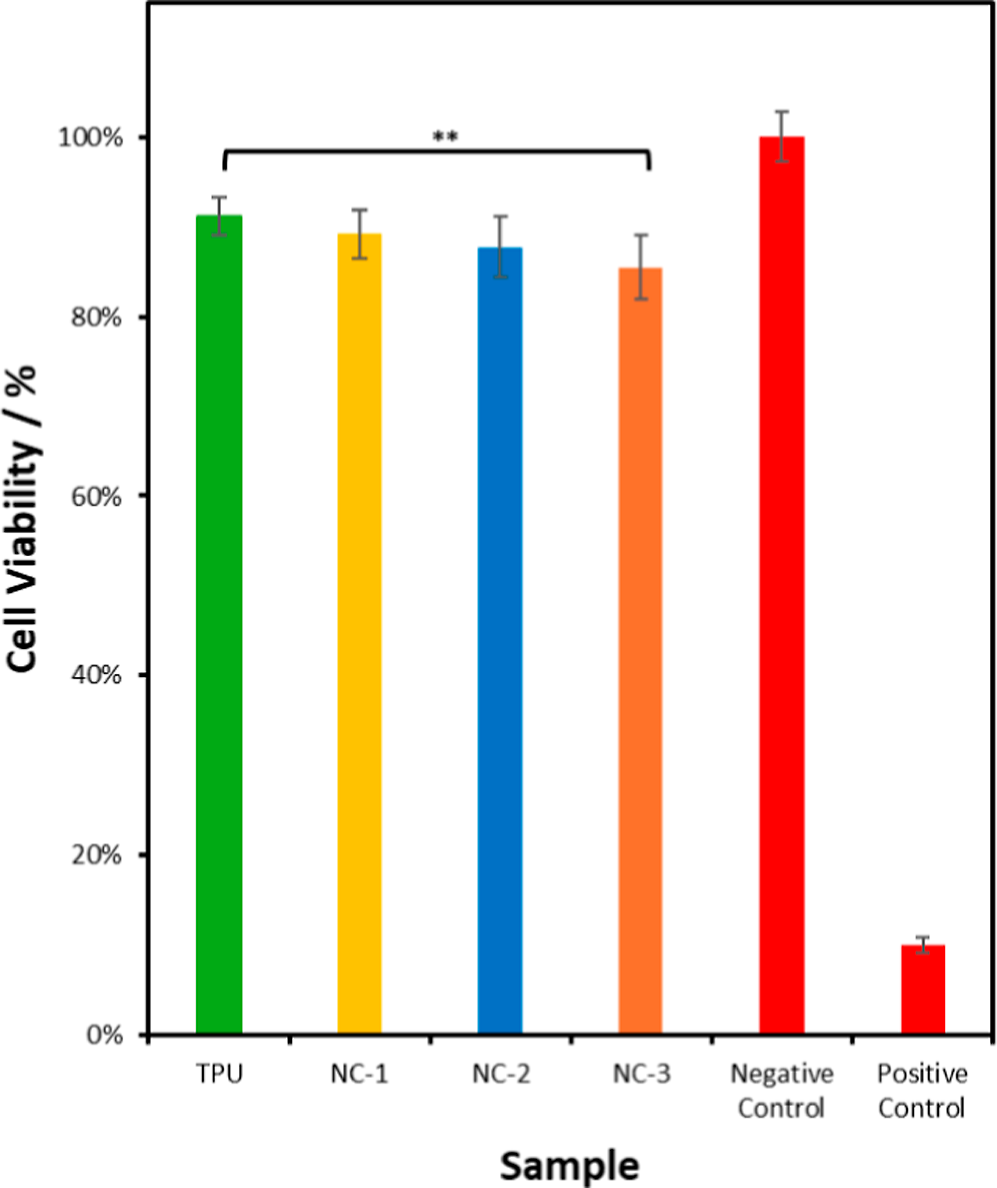
Cytotoxic activity of TPU and NCs (*n* = 6, bars stand for the standard deviation of the mean; **p <0.05 compared with TPU).

#### 3.4.3 Antibacterial Properties

Antibacterial property is another important property for gastrointestinal tissue scaffolds due to them being involved in removing waste from the body, therefore being exposed to high amounts of bacteria in use. The antibacterial properties of TPU, NC-1, NC-2, and NC-3 were evaluated when exposed to *S. aureus* and *E. coli* (Figure 15). Results show that the increased levels of MMT-U in the nanocomposites resulted in increased antibacterial activity against both Gram-positive and Gram-negative bacterial strains. Relative to the TPU sample, NC-1, NC-2, and NC-3 reduce the bacteria by 26%, 39%, and 50% for *S. aureus* and by 24%, 44%, and 59% for *E. coli*, respectively. The antibacterial efficacy can be attributed to the long hydrophobic tails on the surfactant of the MMT-U filler disrupting the bacterial membranes and killing the bacteria.^27^

These results demonstrate that these materials could be suitable for applications in medical devices, tissue repair, and wound dressings.

**Figure 15:**
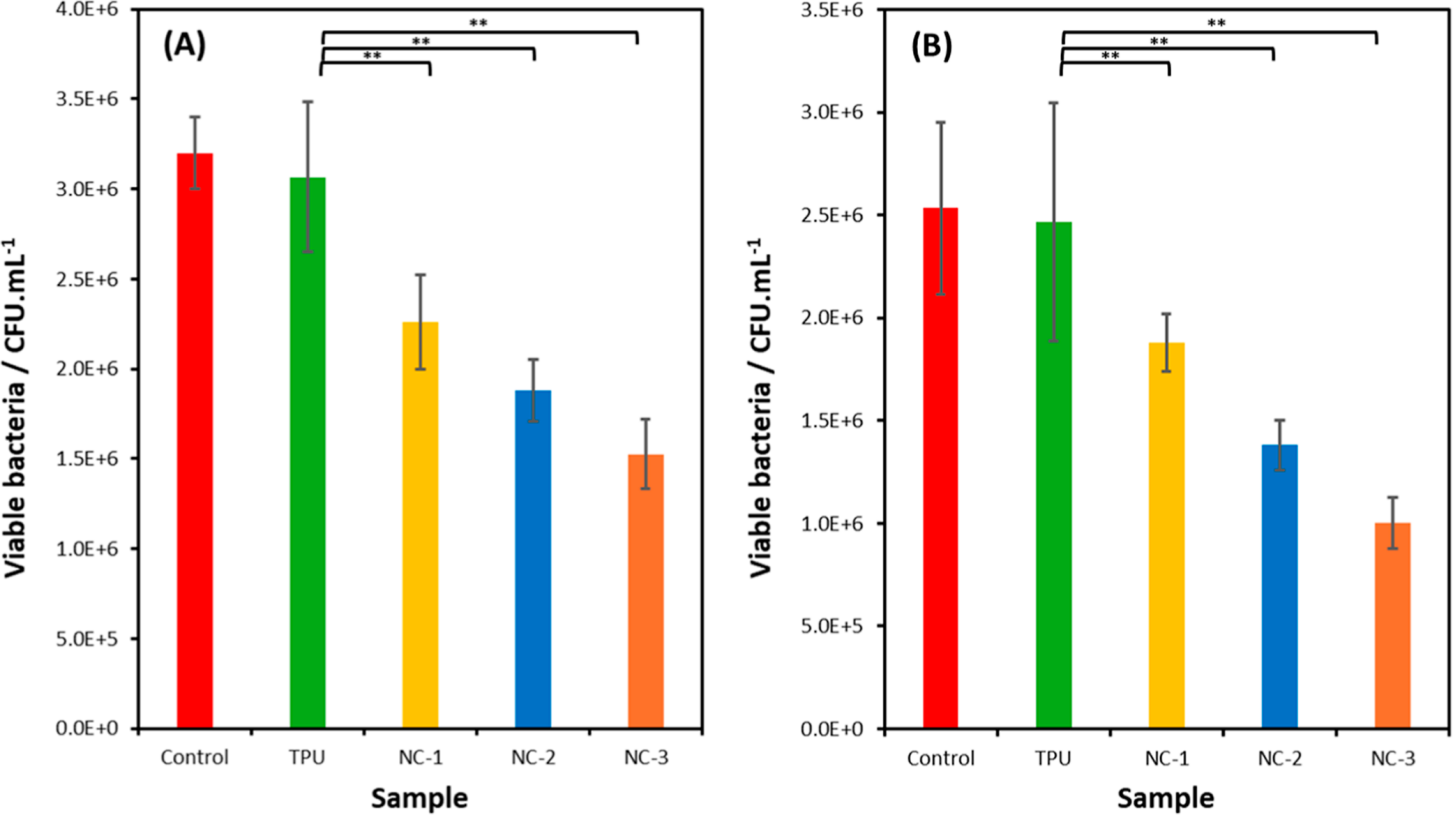
Antibacterial activity against (A) *S. aureus* and (B) *E. coli* (*n* = 3, bars stand for the standard deviation of the mean; **p <0.05 compared with TPU).

#### 3.4.4 Proof-of-Concept Manufacture of Fibrous Tissue Scaffolds

TPU-45 was further processed into fibers to test the materials potential in producing scaffolds for aligned fibrous scaffolds for gastrointestinal soft tissue that shows aligned and/or fibrous microstructure. It can be seen in Figure 16 that fibers produced by wet spinning give a high degree of alignment, a desirable property in the production of fibrous tissue scaffolds. The average diameter of the fibers is 18.65 ± 6.03 μm. While the fiber surface looks smooth, the hydrophilicity of the material could facilitate cell adhesion.^25^

**Figure 16:**
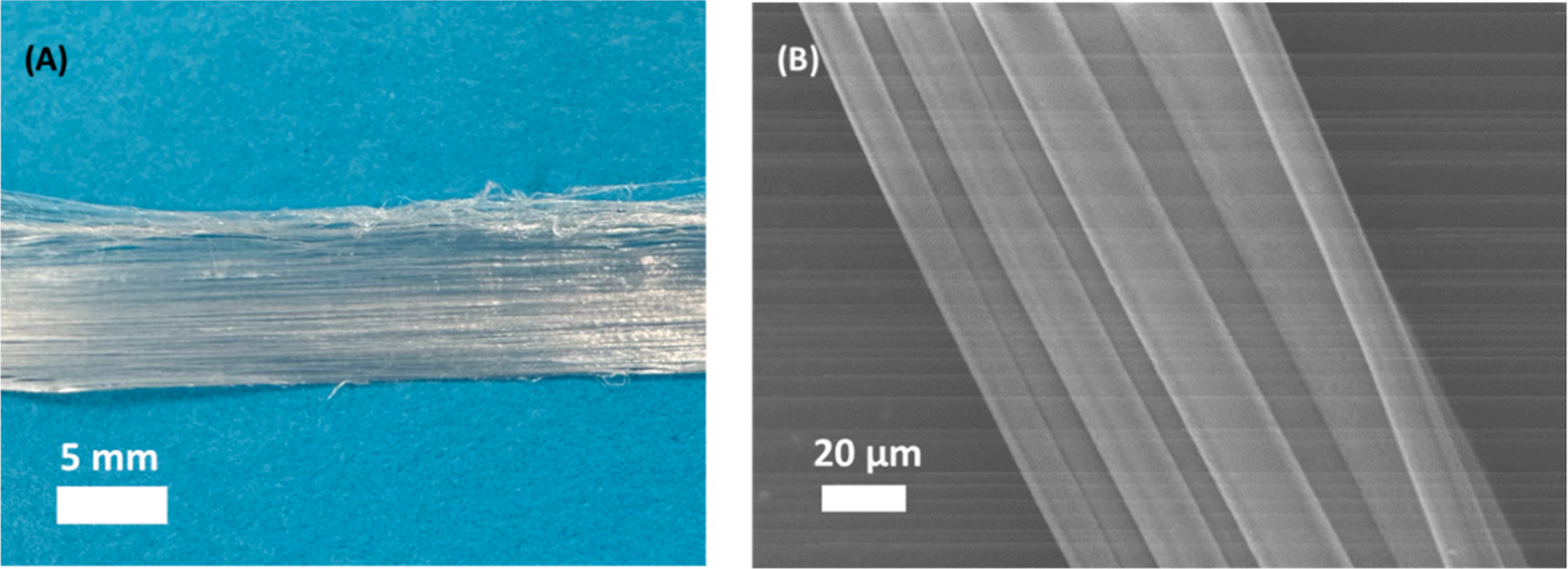
(A) Photograph and (b) SEM image of wet spun TPU-45 fibers.

Unidirectional TPU fiber yarns have a 1.26 ± 0.08 MPa tensile strength and 967 ± 60% strain at break (Figure 17). This is a 194% increase in tensile strength and 70% reduction in strain at break, compared to TPU raw material, which was expected. As the TPU is drawn into fibers the polymer chains become more aligned increasing the tensile strength and reducing the elongation at break.^60^ This elongation at break is comparable to the value of commercial elastane fibers that is typically between 400–500%.^61^ Biobased TPU microfibers with diameters 3.31 ± 1.49 μm reported in literature^24^ provided a higher tensile strength (3.45 ± 0.24 MPa) yet lower elongation at break (302 ± 22%) compared to the TPU fibers in this work. The smaller fibers allowed for more alignment of polymer chains leading to the higher tensile strength and lower elongation at break. The diameter of these fibers could be reduced by the use of a higher gauge needle to inject a narrower jet into the precipitation bath or by adding an additional stretching phase when collecting the fibers. This could lead to a higher tensile strength, with polymer chain alignment being increased further in the smaller diameter.

**Figure 17:**
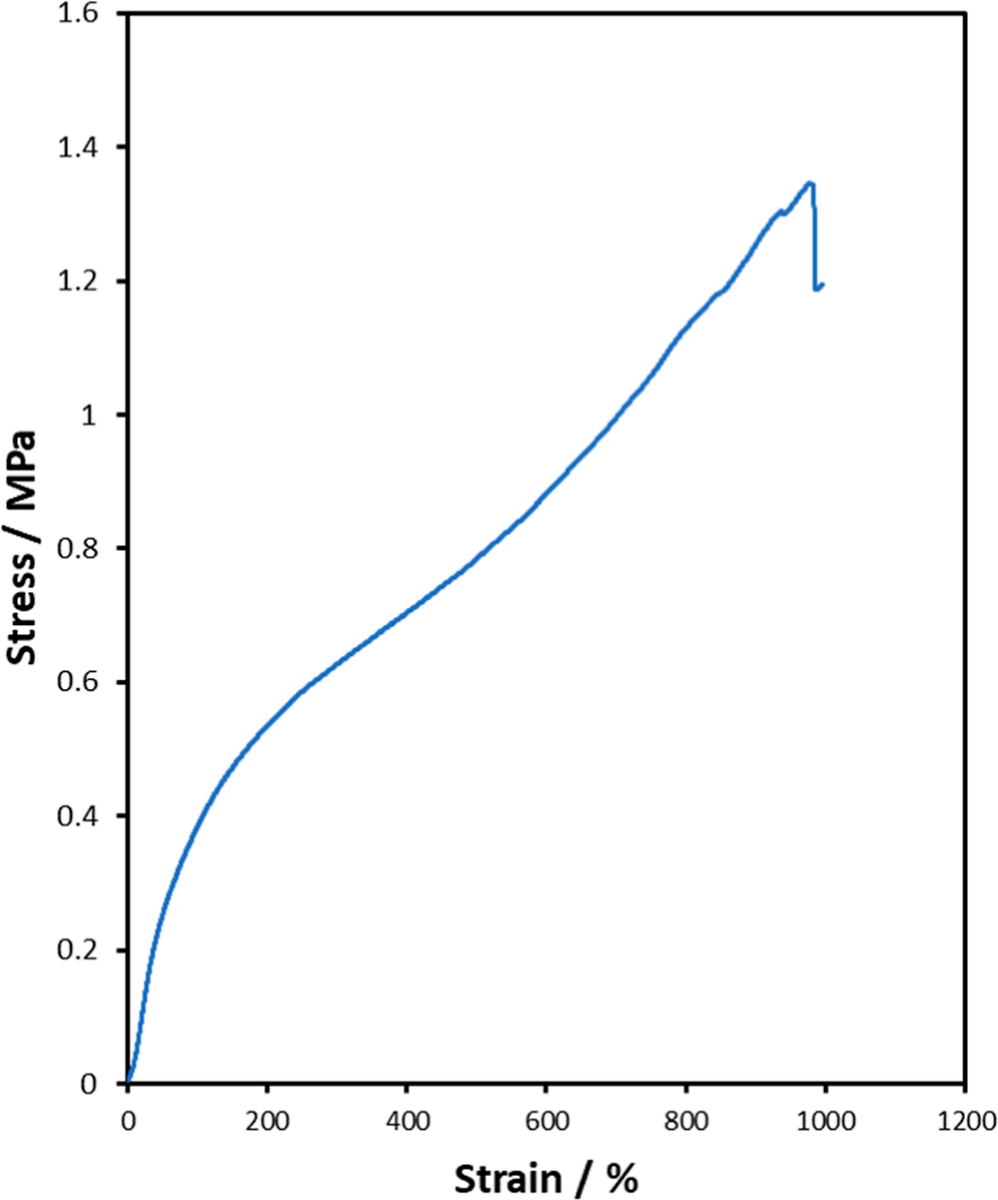
Representative tensile stress–strain curve of spun TPU-45 fibers.

## 4 Conclusions

New amino acid-based TPUs with varying hard segment percentages (40%, 45%, and 50% by molar number) were successfully prepared with the use of a disulfide-containing PCL-diol, LDI, and CHDM. The chemical structure and molecular weight of these TPUs were confirmed with the use of FTIR, NMR, and GPC. DSC and TGA show that the polymers were amorphous having a *T*_g_ of −33.6 °C, −29.1 °C, and −24.3 °C for TPU-40, TPU-45, and TPU-50, respectively, as well as having high thermal stability with all TPUs that can enable melt processing methods where needed. Tensile testing shows that Young’s modulus, yield strength, and tensile strength remain at similar values with increased hard segment ratios. TPU-40 presented the highest strain at break, with TPU-45 and TPU-50 having similar strain at break values. Water contact angles of TPU-40, TPU-45, and TPU-50 became increasingly hydrophilic with the decrease of the hydrophobic PCL ratio in each TPU at 87.7 ± 2.8°, 82.2 ± 0.5°, and 80.4 ± 1.3°, respectively. This combined with Young’s modulus of 0.19 ± 0.01 MPa meant that TPU-45 was best suited for applications in soft tissue repair and further exploration into the use of modified clay to produce bioactive nanocomposites.

Functionalized MMT was produced with the use of 2-undecylimidazoline as the surface modifier. FTIR and XRD confirmed the successful cation exchange reaction between MMT and 2-undecylimidazoline.

TPU-NCs with MMT-U weight percentages (1%, 2%, and 3%) were prepared by mixing them into the TPU-45. Their tensile strength and modulus generally increased with an increased MMT-U content. It was also seen that the yield stress generally increased with MMT-U content; however, the yield strain decreased. Water contact angles of NC-1, NC-2, and NC-3 initially improved but became less hydrophilic with the increased clay content at 78.4 ± 0.1°, 80.5 ± 0.92° and 82.8 ± 0.4°, respectively.

Cytotoxicity tests showed the TPU and NCs are nontoxic. With the inclusion of a higher content of MMT-U, there is a minor reduction in the cell viability that remains above an acceptable level, likely due to the presence of a higher amount of 2-undecylimidazoline in NC-3. Incorporating MMT-U into TPU nanocomposites at low and higher loadings significantly enhances their antibacterial efficacy against both *S. aureus* and *E. coli*, attributable to the long hydrophobic tails on the surfactant of MMT that disrupt and destroy the bacterial cell membranes. These results show that the NCs are both cytocompatible and antibacterial, though there is a trade-off between the cell viability and antibacterial efficacy.

TPU-45 was successfully processed into fibers using a wet spinning process, creating uniform fibers with no beading that reached 1.26 ± 0.08 MPa tensile strength and 967 ± 60% strain at break.

Overall, the results of this research suggest that TPUs, TPU-NCs, and TPU fibers have great potential in the repair of various soft tissue types such as urinary bladder, small intestine, and/or colon. Additional tests on the TPU, MMT-U, and TPU-NCs to understand the biodegradation behavior, cell adhesion, and ability to regenerate resected bowel tissue lost or damaged in ostomy procedures may be conducted. The TPUs and NCs may also be modified to mimic the properties of other soft tissues, for example, in pelvic floor repair.

## Supplementary Material



## Data Availability

The data set underpinning this article will be available in an open-access data repository, Elements, with a description of this article title and DOI.

## Notes

TPU composition and PCL-DS GPC curve (PDF)
