# Supplementary material for: Synthesis and Characterization of Biomimetic Thermoplastic Polyurethanes and Nanocomposites with l‑Lysine Diisocyanate
Source: Biomacromolecules. 2025 Dec 1;27(1):350–64. doi: 10.1021/acs.biomac.5c01488 (PMC12801311; doi:10.1021/acs.biomac.5c01488)
Supplement: Supplementary file 1 [file bm5c01488_si_001.pdf]

Supporting Information

Synthesis and characterisation of biomimetic thermoplastic polyurethanes and nanocomposites with l-lysine diisocyanate

Charlie Bateman[1,2], Chenghao Yao[3], Jingyang Lin[3], Shuai Zhang[3], Biqiong Chen*[1,2]

[1]Department of Chemistry, University of Liverpool, Crown Street, Liverpool, L69 7ZD, United Kingdom

[2]School of Mechanical and Aerospace Engineering, Queen's University Belfast, Stranmillis Road, Belfast, BT9 5AH, United Kingdom

[3]School of Pharmacy, Queen's University Belfast, Lisburn Road, Belfast, BT9 7BL, United Kingdom

**Corresponding author: biqiong.chen@liverpool.ac.uk*

Table S1. Molar ratios of TPU composition

TPU	PCL-DS diol	LDI	CHDM
**TPU-40**	1	4.95	3.50
**TPU-45**	1	5.78	4.25
**TPU-50**	1	6.60	5.00

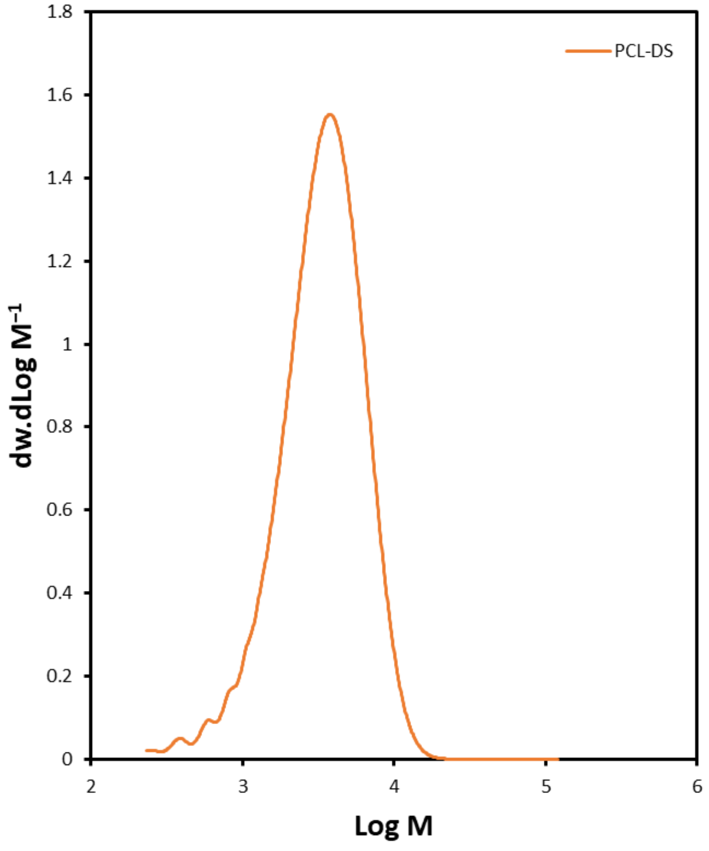

Figure S1. GPC curve for PCL-DS diol
